# Interplay of Tetrel, Hydrogen, and Halogen Bonds in F_3_GeOCl and HCN Complexes: A Comprehensive Theoretical Study of Dimers, Trimers, and Tetramers

**DOI:** 10.1021/acs.jpca.4c08102

**Published:** 2025-01-28

**Authors:** Antonio Frontera, Saeedreza Emamian

**Affiliations:** † Department of Chemistry, 16745 Universitat de les Illes Balears, Crta de Valldemossa km 7.5, 07122 Palma de Mallorca, Baleares, Spain; ‡ Department of Chemistry and Biochemistry, Shahrood Branch, Islamic Azad University, 36714 Shahrood, Iran

## Abstract

This study investigates the nature and interplay of noncovalent interactions (NCIs)—tetrel bonds (TB), hydrogen bonds (HB), and halogen bonds (XB)—in molecular assemblies formed between trifluorogermyl hypochlorite (F_3_GeOCl) and hydrogen cyanide (HCN). Using a combination of high-level computational methods, we explored the geometric, energetic, and electronic properties of dimers, trimers, and tetramers formed in different molar ratios of interacting reagents. Various analyses reveal a significant cooperativity between TB and HB, which mutually reinforce each other, while XB interactions are diminished in the presence of TB and HB. Energy decomposition analysis (EDA) through SAPT and sobEDAw methods identified electrostatic and orbital interactions as key contributors to the stabilization of TB and HB, while dispersion plays a prominent role in XB. A perfect linear correlation was found between interaction energy and charge density at bond critical points (BCPs), underscoring the predictive value of these metrics. These findings shed light on the cooperative nature of NCIs and provide a framework for designing molecular systems in supramolecular chemistry and crystal engineering.

## Graphical abstract

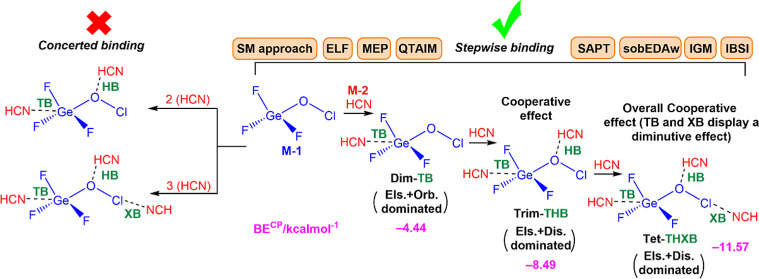

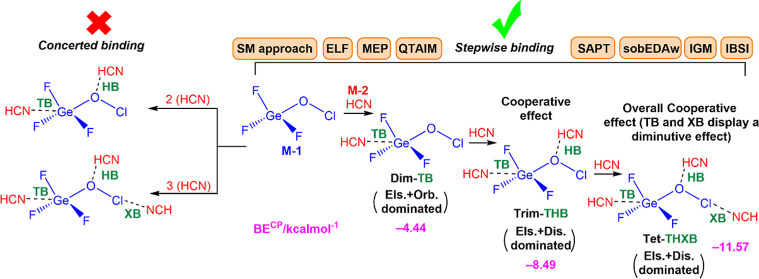

## 1 Introduction

Generally, a significant portion of chemistry can be attributed to interactions between Lewis acids (LAs) and Lewis bases (LBs). However, not all such interactions result in chemical reactions. Noncovalent interactions (NCIs), also referred to as closed-shell interactions, are one of the most important classes of interactions, playing crucial roles in a wide range of physical, chemical, and biological systems.^1^ For instance, NCIs significantly influence the structure and stability of molecular assemblies and crystals.^2^ Furthermore, processes such as drug binding, protein folding, self-assembly, nucleobase stacking, and crystal packing are direct outcomes of NCIs.^3^ Given their critical roles, it is unsurprising that NCIs are receiving increasing attention in various fields.^4–6^

Hydrogen bonds (HBs) are among the strongest and most recognized noncovalent interactions (NCIs), occurring both inter and intramolecularly.^7–10^ IUPAC defines HBs as attractive interactions between a hydrogen atom, covalently bound to a more electronegative atom, and another atom or group acting as electron donor.^9^ This definition can be extended to other NCIs, which can be described through “hole–lump” interactions. The “σ-hole” concept, introduced by Politzer et al.,^10^ explains how halogen atoms, despite being electronegative, can act as electron acceptors in halogen bonds (XBs) due to anisotropic electron density distribution. This phenomenon, known as “polar flattening”,^11^ gives halogens both nucleophilic and electrophilic character, as illustrated in molecular electrostatic potential (MEP) maps.^10–12^ This positive region along the R–X bond is termed the “σ-hole.” The polar flattening phenomenon that creates this positive region extends to other atoms, explaining the formation of different types of NCIs (as shown in Scheme 1) via the attractive interaction between the σ-hole and the negative region of a Lewis base (LB).^13^ When atom Y from groups III–VII is covalently bonded to an electron-withdrawing group (R), forming a sufficiently positive σ-hole, it can engage in various NCIs, such as “triel bonds (TrB),” “tetrel bonds (TB),” “pnictogen bonds (PnB),” “chalcogen bonds (ChB),” and “halogen bonds (XB)”.^14,15^ Although electrostatic interactions primarily stabilize these σ-hole NCIs, dispersion forces and charge transfer may also play a role.^16–18^ Note that even hydrogen bonds (HBs) have been proposed to follow the σ-hole model.^19^ Additionally, heavier elements from the same group tend to create stronger NCIs due to increased polarizability, leading to the more positive σ-holes when bound to electron-withdrawing groups.^20^ For example, in F–Cl, F–Br, and F–I, the most polarizable I atom creates the strongest σ-hole, resulting in the most robust XB interaction with ammonia as the LB.

**Scheme 1:**
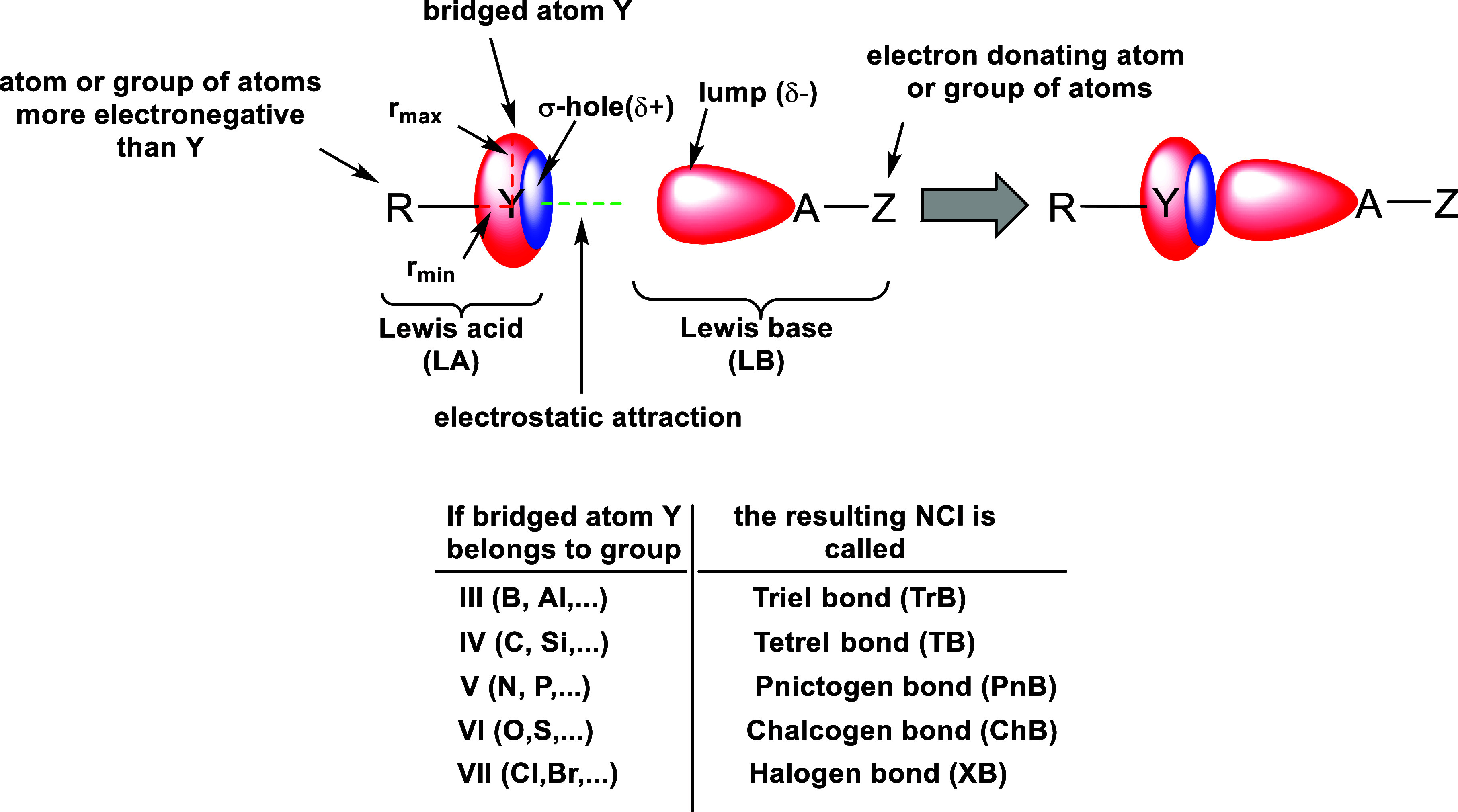
Formation of an NCI between an LA (Electron Acceptor) R–Y and an LB (Electron Donor) A–Z Based on the “Hole–Lump” Concept^a^

a The type of the resulting “σ-hole” NCI is determined by the type of bridged atom Y. A is an atom bearing LP such as N, O, S, etc. See text for details.

Cooperativity (nonadditivity) is a key concept in understanding NCIs.^21^ When an NCI links two molecules, their potential to form further NCIs is influenced by mutual polarization, leading to effects like shorter binding distances, increased interaction energy, and shifts in vibrational modes involved in the interaction.^22,23^ Many studies explored cooperativity in NCIs, such as in ChB,^24^ TB,^25^ and XB^26^ chains. Additionally, cooperativity between different σ-hole NCIs has been examined. For instance, Li et al. investigated the cooperative effect between PnB and XB in assemblies like XCl···FH_2_···NH_3_ and found that XB interactions exhibit stronger cooperativity.^27^ Cooperativity in a trimer (A···B···C) can strengthen both interactions (synergism, negative cooperative energy *E*_coop_), weaken both (antagonism, positive *E*_coop_), or strengthen one while weakening the other, usually resulting in near-zero *E*_coop_ values.^28–31^

In this study, as shown in Scheme 2, we use ab initio quantum calculations to explore the interactions between trifluorogermyl hypochlorite (F_3_GeOCl, monomer **M-1**) and hydrogen cyanide (HCN, monomer **M-2**).

**Scheme 2:**
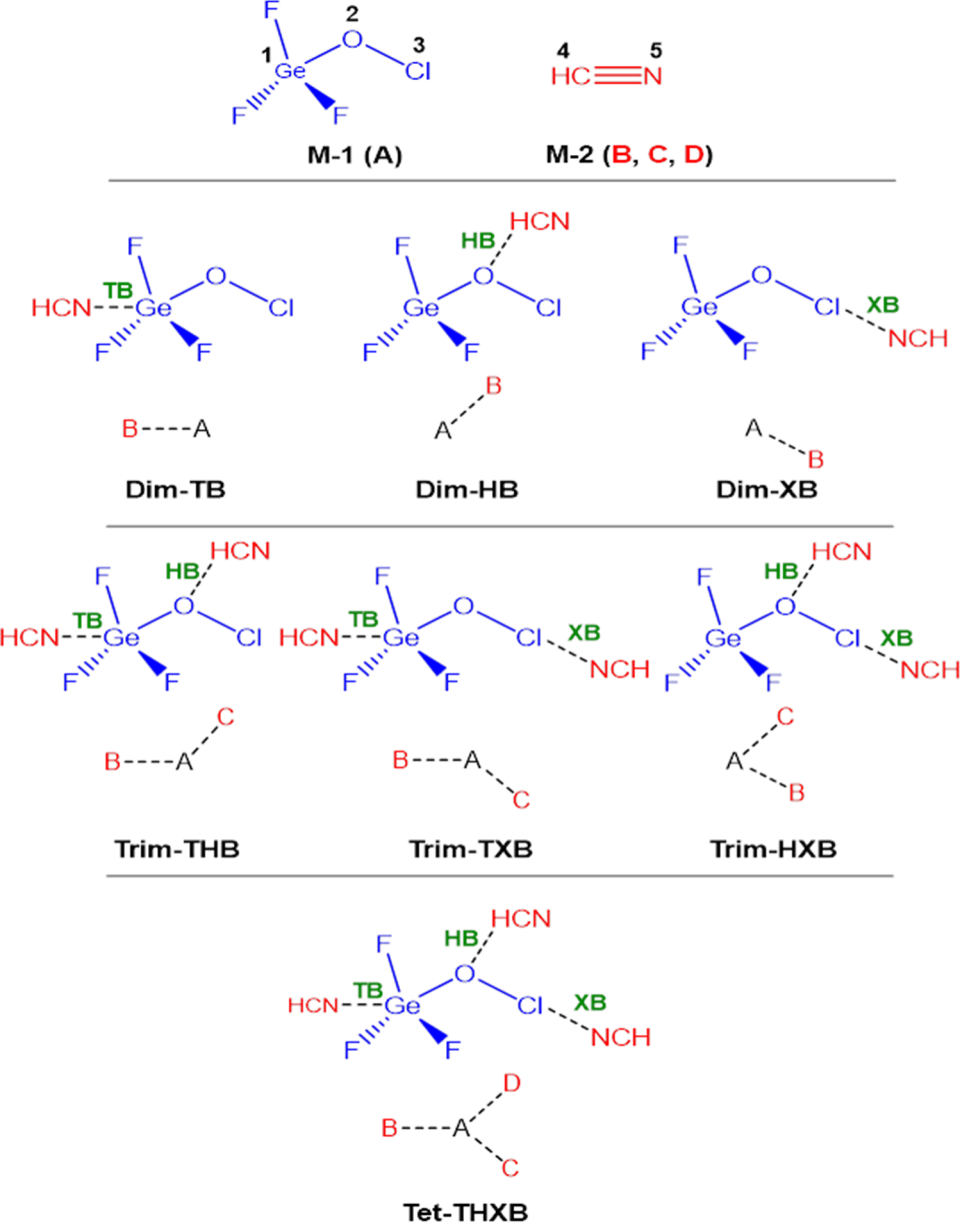
Structure of the Investigated Dimers (**Dim-TB**, **Dim-HB**, and **Dim-XB**), Trimers (**Trim-THB**, **Trim-TXB**, and **Trim-HXB**), and the Only Feasible Tetramer (**Tet-THXB**) Which Can Form through NCI between **M-1** and **M-2** ^a^

a TB = tetrel bond, HB = hydrogen bond, XB = halogen bond, THB = tetrel with hydrogen bond, TXB = tetrel with halogen bond, HXB = hydrogen with halogen bond, and THXB = tetrel, hydrogen, and halogen bond together.

The focus is on understanding the electronic nature, strength, and formation mechanisms of the NCIs that emerge, particularly their competition and interplay (cooperativity, noncooperativity, or anticooperativity). In **M-1**, Ge is covalently bound to three highly electronegative F and one O atoms. Moreover, the Cl atom is covalently bound to the O atom. Therefore, **M-1** features sufficiently positive σ-holes on both the Ge and Cl atoms, allowing for tetrel bond (TB) and halogen bond (XB) interactions with the lone pair (LP) on the N atom of **M-2** as a common nitrogen-based LB considered in the NCIs studies. Additionally, the O atom in **M-1** can form hydrogen bonds (HB) with the H atom of **M-2**. Depending on the molar ratio of the two monomers, different assemblies may form: dimers (**Dim-TB**, **Dim-HB**, **Dim-XB**) at a 1:1 ratio, trimers (**Trim-THB**, **Trim-TXB**, **Trim-HXB**) at a 1:2 ratio, and a tetramer (**Tet-THXB**) at a 1:3 ratio. This study offers a detailed view of how these NCIs drive molecular assembly.

## 2 Computational Details

A full geometry optimization followed by a frequency calculation was performed over the structures given in Scheme 2 utilizing second order Møller–Plesset (MP2) perturbation method^32^ in conjunction with aug-cc-pVDZ basis set^33,34^ by means of Gaussian 16 revision A.03 ^35^ software program package. The MP2/aug-cc-pVDZ computational level exhibits reliable results and, is frequently employed to study various kinds of NCIs.^36^ In addition, the results obtained with the aug-cc-pVDZ basis set is demonstrated to be comparable with those generated through the quantum gold method namely coupled-cluster calculations with single, double, and perturbative triple excitations, CCSD­(T), in conjunction with higher basis sets.^37–39^ The absence of imaginary frequencies demonstrates that all optimized structures (monomers, dimers, trimers, and tetramer given in Scheme 2) are true minima located over the potential energy surface (PES) of the complexation processes.^40^

For a given complexation process e.g., A + B → A···B, raw interaction energy (IE^raw^) as a measure of the A···B interaction strength could simply be estimated through supermolecular (SM) approach by the subtracting of the sum total electronic energy of monomers A and B from the total electronic energy of dimer A···B, all frozen in the fully optimized structure of dimer A···B.^41^ The very accurate value of interaction energy corrected for basis set superposition energy error (*E*_BSSE_), based on the counterpoise (CP) methodology provided by Boys and Bernardi,^42^ and extrapolated toward complete basis set (CBS) limit was evaluated through the well-known “focal point” technique^43,44^ for any complexation processes as

1 $${\text{IE}}^{\text{CP}}={\left(\Delta E_{\text{HF}}^{\text{SCF}}+\Delta E_{\text{MP}2}^{\text{corr}}\right)}_{\text{aug}‐\text{cc}‐\text{pV}\left[\text{DT}\right]Z}+\Delta {\left(E_{\text{CCSD}\left(T\right)}^{\text{corr}}-E_{\text{MP}2}^{\text{corr}}\right)}_{\text{aug}‐\text{cc}‐\text{pVDZ}}+E_{\text{BSSE}}$$

where, IE^CP^ denotes the counterpoise-corrected interaction energy (IE^CP^ = IE^raw^ + *E*_BSSE_) of the complex formed, Δ*E*_HF_^SCF^ and Δ*E*_MP2_^corr^ represent the self-consistent field and correlation portion of the interaction energy estimated at, respectively, Hartree–Fock (HF) and MP2 methods both extrapolated toward CBS limit using aug-cc-pVDZ and aug-cc-pVTZ basis sets (aug-cc-pVDZ → aug-cc-pVTZ). The next term indicated by a delta notation is the difference in the CCSD­(T) and MP2 correlation energy both of which are calculated taking the smaller aug-cc-pVDZ basis set. This term, indeed, covers the MP2 deficiency in the exact calculation of the correlation energy.^43,44^ The focal point-based computations were carried out by means of PSI4 software program package revision 1.9.1.^45^ For any complexation processes considered, the counterpoise corrected binding energy, BE^CP^, was determined through augmentation of the calculated IE^CP^ value with total geometrical deformation energy; that is

2 $${\text{BE}}^{\text{CP}}={\text{IE}}^{\text{CP}}+\Delta E_{\text{tot}}^{\mathrm{def}}$$

In eq 2, Δ*E*_tot_^def^ stands for the total geometrical deformation energy as the sum of geometrical deformation energy of participating monomers **M-1** and **M2**

3 $$\Delta E_{\text{tot}}^{\mathrm{def}}=\Delta E_{M‐1}^{\mathrm{def}}+\Delta E_{M‐2}^{\mathrm{def}}$$

It should be indicated that the geometrical deformation energy is an energy penalty for geometrically distorting (or, reshaping) a monomer from its equilibrium geometry to the geometry it adopts in the fully optimized complex structure (whole system).^46^ The geometrical deformation energies were calculated at the computational level employed for the geometrical optimizations. Note, however, that for a quite linear and rigid structure such as monomer **M-2** (HCN) whose variable geometrical parameters consist of only one H–C–N angle and two H–C and C–N bond distances the geometrical deformation energy is expected to be negligible.

An accurate prediction of the reactive sites of monomers engaged in a given NCI is made possible using the electrostatic potential, *V*(*r*),^47^ as a fundamental molecular property and a physically observable quantity.^48^ Murray and Politzer^49^ have recommended that the locally most positive region possessing the value of *V*_s,max_ (where the hole is located) in a given electrophilic species (an LA) and the locally most negative region possessing the value of *V*_s,min_ (where the lump is located) in a given nucleophilic species (an LB) which participate in the formation of an NCI is more reasonable and meaningful to be evaluated and analyzed on the 0.001 electrons per bohr^3^ (e/bohr^3^) contour of electron density surrounding the monomer’s molecular electrostatic potential (MEP) map. To this end, the MP2/aug-cc-pVDZ generated monodeterminantal wave function of the fully optimized monomers **M-1** and **M-2** was taken into account to compute values of *V*_s,max_ and *V*_s,min_ and sketch 3-D MEP isosurfaces employing WFA-SAS suite of program.^50^

The MP2/aug-cc-pVDZ generated monodeterminantal wave function of the fully optimized complexes was also taken into account to perform the Bader’s quantum theory of atoms in molecules (QTAIM)^51^ analysis by means of AIMAll version 19.10.12 (professional) software package.^52^ The QTAIM analysis characterizes key topological descriptors at the bond critical point (BCP) located along a bond path connecting two interacting atoms during the formation of NCIs studied.

The electron density-based descriptors namely independent gradient model (IGM)^53^ and intrinsic bond strength index (IBSI)^54^ proposed by Lefebvre and co-workers are able to figure out both noncovalent and covalent interactions in a quite apparent and separate manner and, represent a numerical scale for the strength of various interactions, respectively. Using the MP2/aug-cc-pVDZ generated monodeterminantal wave function of the fully optimized complexes, IGM analysis together with drawing corresponding 2-D plots and evaluation of IBSI value were performed for various NCIs in the complexes investigated utilizing IGMPLOT 3.08.^55^

Energy decomposition analysis (EDA) was performed utilizing symmetry-adapted perturbation theory (SAPT)^56^ and, also, sobEDA method which has very recently been proposed by Lu and Chen.^57^ PSI4 version 1.9.1 ^45^ was used to perform SAPT analysis at the “gold” SAPT2 + (3)­δ MP2/aug-cc-pVTZ level^58^ while sobEDA calculations were executed employing a specific script^59^ prepared by Lu.

Electron localization function (ELF)^60^ analyses were carried out using the MP2/aug-cc-pVDZ monodeterminantal wave function of the fully optimized target structures by means of TopMod program^61^ and, corresponding 3-D isosurfaces were sketched using VMD 1.9.1 visualizing software.^62^

## 3 Results and Discussion

This study is structured into five main sections: (i) Section 3.1: based on the “hole–lump” concept introduced earlier, we identify the positions of holes and lumps in monomers **M-1** and **M-2** to explain their potential involvement in various NCIs. This is achieved through ELF analysis and the evaluation of the Laplacian of electron density (∇^2^ρ) to pinpoint these regions; (ii) Section 3.2: the MEP maps of the monomers are thoroughly analyzed to determine the locations of maximum and minimum electrostatic potential, which play key roles in the formation of NCIs, reflecting the electrostatic nature of the hole–lump interactions; (iii) Section 3.3: this section delves into the energetics and PES of the complexation processes to clarify the composition of reaction mixtures and the mechanisms behind the formation of dimers, trimers, and tetramers (Scheme 2). The impact of NCIs is also examined through the lens of cooperativity; (iv) Section 3.4: we track and analyze the signature of the various NCIs identified, focusing on geometric changes during complexation, topological descriptors at bond critical points (BCPs), and IGM and IBSI values to achieve the section’s objectives. These findings are then compared to the energetic analyses to confirm mutual influences among the NCIs; (v) Section 3.5: the final section conducts an EDA to determine the electronic nature of the NCIs and to identify the intermolecular forces most responsible for stabilizing the complexes formed between the monomers.

### 3.1 Analysis of Hole and Lump in Monomers **M-1** and **M-2**

An ELF analysis and a QTAIM calculation was carried out on the MP2/aug-cc-pVDZ monodeterminantal wave function of the fully optimized structure of **M-1** and **M-2** and, corresponding results are portrayed in Figure 1. In the Bader’s QTAIM^51^ it is well documented that accumulation of the charge density (ρ) at a given point (such as at the BCP of a covalent bond or at the position associated with the location of LPs called lump) is characterized with a negative value of Laplacian of charge density, ∇^2^ρ < 0. On the other hand, depletion of charge density (such as at the hole position) is distinguished with a positive value of Laplacian of charge density, ∇^2^ρ > 0.

**Figure 1:**
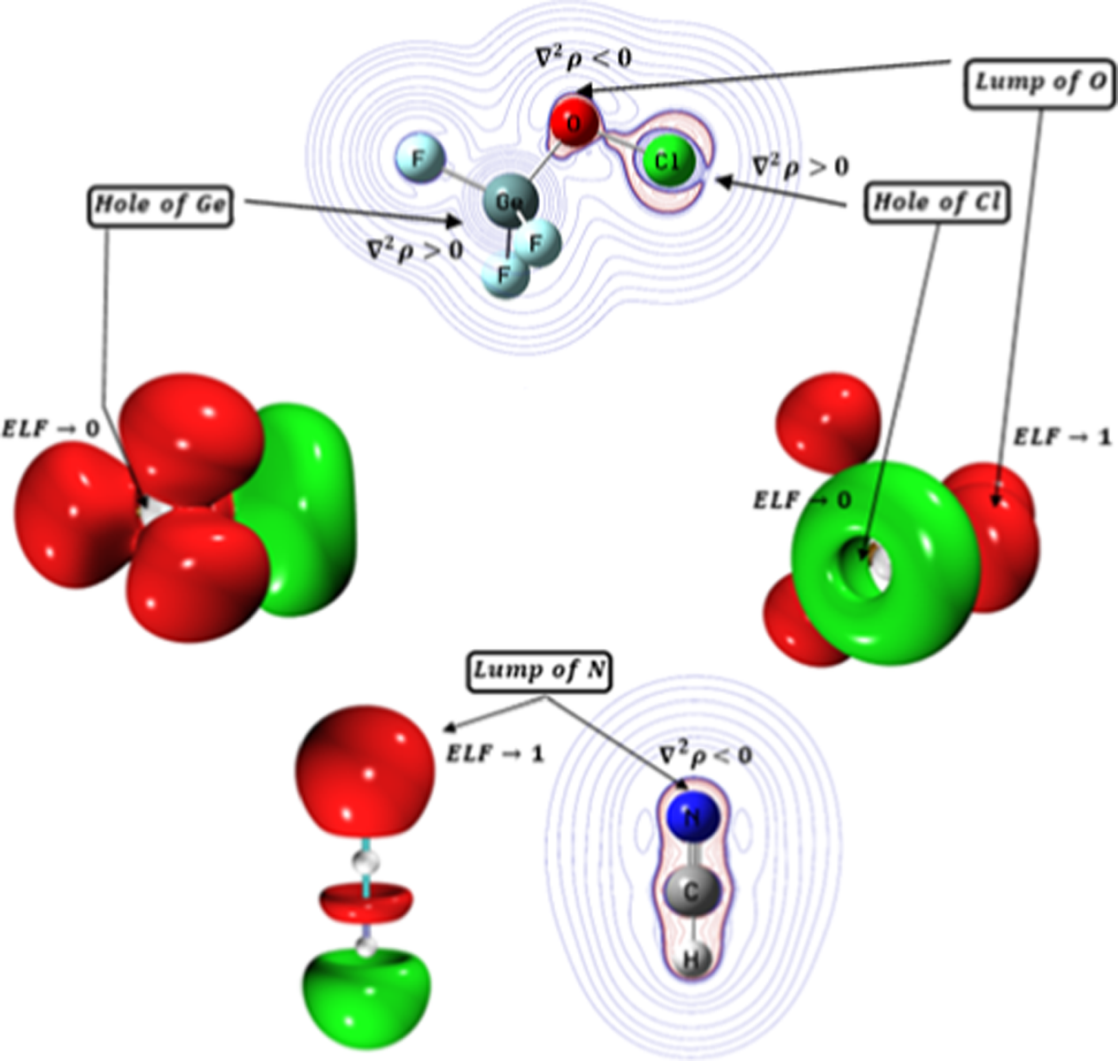
Position of “hole” where ∇^2^ρ is positive, ∇^2^ρ > 0 (blue contour lines), and ELF approaches its minimum value, ELF → 0, and of “lump” where ∇^2^ρ is negative, ∇^2^ρ < 0 (red contour lines), and ELF approaches its maximum value, ELF → 1, in the MP2/aug-cc-pVDZ fully optimized (top) monomers **M-1** (GeF_3_OCl) and (bottom) monomer **M-2** (HCN). An isovalue of 0.800 and 0.001 au was used to sketch 3-D ELF isosurface and 2-D contours of ∇^2^ρ, respectively. Monosynaptic basins are shown in green and disynaptic basins are shown in red.

The ELF is a key measure for determining the degree of electron localization within a given region, with values ranging from 0 (minimum) to 1 (maximum).^60^ Along with the sign of ∇^2^ρ, ELF provides two important descriptors for identifying whether a point in the AIM space of an atomic or molecular assembly corresponds to a hole or a lump. As shown in Figure 1, the presence of a Ge σ-hole along the O–Ge bond extension and behind the Ge atom, as well as a Cl σ-hole along the O–Cl bond extension on the crown of the Cl atom in **M-1**, is indicated by blue ∇^2^ρ contour lines, where ∇^2^ρ is positive.

These holes are further confirmed by the ELF 3-D isosurface, where ELF approaches 0, indicating minimal electron localization in these regions. In contrast, a lump associated with the oxygen lone pairs (LPs) in **M-1** is identified by red ∇^2^ρ contour lines around the O atom (where ∇^2^ρ is negative), and by the ELF 3-D isosurface where ELF approaches 1 (ELF → 1), indicating a high degree of electron localization. A similar lump corresponding to the nitrogen LP of **M-2** is also shown in Figure 1. Therefore, the Ge σ-hole and Cl σ-hole in **M-1** can interact with the nitrogen lump in **M-2**, leading to the formation of a Ge···N tetrel bond (TB) and a Cl···N halogen bond (XB). Additionally, the oxygen LP in **M-1** can interact with the acidic H atom in **M-2** (bound to the electron-withdrawing CN group), forming an O···H hydrogen bond (HB). Thus, the NCIs studied in this investigation include three intermolecular hole–lump interactions (Ge···N TB, Cl···N XB and O···H HB).

### 3.2 MEP Maps of Monomers **M-1** and **M-2**

Within the “hole–lump” concept, the attractive interaction between two monomers is rationalized through electrostatic attraction between the most positive region over the MEP map of the electrophilic monomer (LA), representing a hole where *V*_s,max_ is located, and the most negative region over the MEP map of the nucleophilic monomer (LB), representing a lump where *V*_s,min_ is located. Employing the MP2/aug-cc-pVDZ monodeterminantal wave function of the fully optimized structure of **M-1** and **M-2**, the value of *V*_s,max_ associated with the maximum electrostatic potential for atoms Ge1 and Cl3 of **M-1** and atom H4 of **M-2** together with the value of *V*_s,min_ associated with the minimum electrostatic potential for atom O2 of **M-1** and atom N5 of **M-2** was estimated by means of the WFA-SAS software and corresponding numerical values as well as 3-D isosurfaces are illustrated in Figure 2. As evidently given in Figure 2, the maximum electrostatic potential regarding the MEP surface surrounding Ge1 and Cl3 atoms of **M-1** is located at the σ-hole position of these atoms with a significant *V*_s,max_ value of 52.45 and 34.50 kcal·mol^–1^, respectively. On the other hand, the minimum electrostatic potential regarding the MEP surface surrounding O3 atom of **M-1** is located at its lump position representing a *V*_s,min_ value of −6.23 kcal·mol^–1^. However, the maximum and minimum electrostatic potential corresponding to the MEP surface surrounding H4 and N5 atoms of **M-2** is located, respectively, at H4 atom, *V*_s,max_ = 51.65 kcal·mol^–1^, and at the lump position associated with the LP of N atom, *V*_s,min_ = −31.45 kcal·mol^–1^.

**Figure 2:**
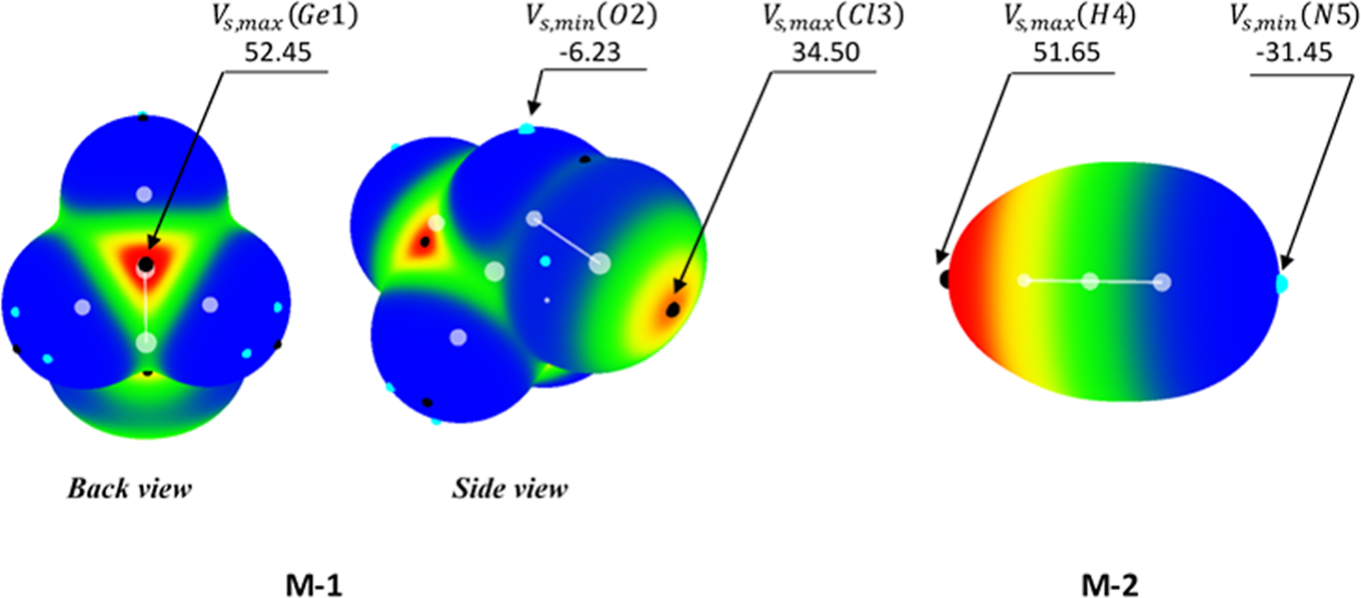
MP2/aug-cc-pVDZ 3-D representation of MEP mapped on the electron density isosurface of 0.001 e/bohr^3^ together with the position as well as the value of *V*_s,max_ and *V*_s,min_, in kcal·mol^–1^, for the reactive sites in the fully optimized monomers **M-1** and **M-2**. The black and cyan circles over the 3-D MEP isosurfaces characterize the position of positive and negative regions, respectively. Atom numbering is given, as well.

Therefore, during the formation of Ge···N TB or Cl···N XB, the lump of N in **M-2** approaches the hole of Ge or the hole of Cl in **M-1**, respectively. The formation of the O···H HB also takes place when the maximum electrostatic region of H in **M-2** approaches the minimum electrostatic region of O in **M-1**. Taking the absolute difference between values of *V*_s,max_ and *V*_s,min_ evaluated for the reactive sites in **M-1** and **M-2** and *as far as electrostatic interactions are concerned*, one expects that the stability order of dimers formed by the interactions between **M-1** and **M-2** (see Scheme 2) to be as **Dim-TB** > **Dim-HB** > **Dim-XB**.

### 3.3 Energetics Analysis and Mechanistic Exploration

As shown in Scheme 2, dimer formation occurs when **M-1** and **M-2** interact in a 1:1 ratio. This leads to the formation of **Dim-TB** (with one Ge···N TB), **Dim-HB** (with one O···H HB), or **Dim-XB** (with one Cl···N XB). For trimers, an interaction between one molecule of **M-1** and two molecules of **M-2** is required. This results in the formation of **Trim-THB** (with Ge···N TB and O···H HB), **Trim-TXB** (with Ge···N TB and Cl···N XB), or **Trim-HXB** (with O···H HB and Cl···N XB). Lastly, a 1:3 ratio of **M-1** to **M-2** leads to the formation of the tetramer **Tet-THXB**, which simultaneously includes all three types of NCIs found in the dimers and trimers. The energetic descriptors for the formation of these complexes from corresponding separate monomers are calculated and presented in Table 1. To clarify, the stability trends discussed in this section and throughout the manuscript specifically pertain to the particular dimers or trimers studied in this work. These trends should not be generalized to other systems or to tetrel bonds (TB), halogen bonds (XB), and hydrogen bonds (HB) interactions in general.

**Table 1:** Computed Value^a^ of raw Interaction Energy, IE^raw^, Basis Set Superposition Energy Error, *E*_BSSE_, Counterpoise-Corrected Interaction Energy, IE^CP^, Geometrical Deformation Energy of Monomer **M-1**, Δ*E*_M‑1_^def^, and of Monomer **M-2**, Δ*E*_M‑2_^def^, Total Geometrical Deformation Energy, Δ*E*_tot_^def^ = Δ*E*_M‑1_^def^ + Δ*E*_M‑2_^def^, Raw Binding Energy, BE^raw^, and Counterpoise-Corrected Binding Energy, BE^CP^, in kcal·mol^–1^, Together with the Presence Percentage, % *P*, of Each Complex within Its Respective Class Evaluated Based on the Boltzmann’s Distribution for the Dimers, Trimers, and Tetramer Investigated (the BE^CP^ Values Were Used for the Calculation of % *P*)

complex	IE^raw^	*E*_BSSE_	IE^CP^	Δ*E*_M‑1_^def^	^b^Δ*E*_M‑2_^def^	Δ*E*_tot_^def^	BE^raw^	BE^CP^	% *P*
**Dim-TB**	–14.04	2.73	–11.31	6.80	0.07	6.87	–7.17	–4.44	61.19
**Dim-HB**	–2.97	1.14	–1.83	0.07	0.00	0.07	–2.90	–1.76	0.66
**Dim-XB**	–4.84	0.55	–4.29	0.13	0.00	0.13	–4.71	–4.16	38.15
**Trim-THB**	–23.29	4.41	–18.88	10.42	0.12	10.54	–12.75	–8.31	82.27
**Trim-TXB**	–13.83	2.75	–11.08	4.06	0.04	4.10	–9.73	–6.98	8.72
**Trim-HXB**	–8.93	1.80	–7.13	0.11	0.02	0.13	–8.80	–7.00	9.02
**Tet-THXB**	–24.14	4.61	–19.53	7.97	0.11	8.08	–16.06	–11.45	100

a While the value of geometrical deformation energy is calculated at the MP2/aug-cc-pVDZ level, other energetics descriptors are extrapolated toward CBS limit employing focal point approach.

b Δ*E*_M‑2_^def^ includes the sum of deformation energy values for all molecules **M-2** in the complexes where more than one molecule of **M-2** is present.

As shown in Table 1, among the dimers formed, the most negative raw interaction energy (IE^raw^) of −14.04 kcal·mol^–1^ corresponds to **Dim-TB**, where a Ge···N tetrel bond forms between the σ-hole of Ge in **M-1** and the LP of N in **M-2**. **Dim-XB** and **Dim-HB** follow with IE^raw^ values of −4.84 and −2.97 kcal·mol^–1^, respectively. When corrected for BSSE, the IE^CP^ values become more positive. Notably, the main source of deformation energy comes from **M-1**, as **M-2** remains largely unchanged during complexation. In particular, **M-1** undergoes significant deformation during **Dim-TB** formation, with a deformation energy Δ*E*_M‑1_^def^ = 6.80 kcal·mol^–1^. This is due to structural changes in the sp^3^-hybridized Ge atom, where F–Ge–O bond angles are significantly altered by the repulsion between the nitrogen LP in **M-2** and LPs of the fluorine atoms in **M-1**.

Once deformation energies are factored in, the binding energies (BE^CP^) show that **Dim-TB** has the largest difference between IE^CP^ and BE^CP^, indicating considerable deformation energy. All dimers show negative BE^CP^ values, confirming their stability relative to the isolated monomers, with the stability order **Dim-TB** ≈ **Dim-XB** ≫ **Dim-HB**. This stability order suggests that factors beyond electrostatic potential contribute to stabilization (discussed later).

For trimers and the tetramer, the trend is similar. All complexes are more stable than their monomers, with the order of stability for trimers being **Trim-THB** > **Trim-HXB** ≈ **Trim-TXB**. The strong TB interaction in **Trim-THB** and **Trim-TXB** results in more negative IE^CP^ values (−18.88 and −11.08 kcal·mol^–1^, respectively), while **Trim-HXB**, lacking a TB, has a less negative IE^CP^ of −7.13 kcal·mol^–1^. However, due to the greater deformation energy in **Trim-TXB**, **Trim-HXB** becomes slightly more stable, highlighting the critical role of deformation in systems with high flexibility.

The % *P* index, shown in the last column of Table 1, indicates the percentage presence of each complex within its respective class. This index is calculated using the Boltzmann contribution function, as expressed by the following equation^63^

4 $$\%\;P=\frac{\exp\left(\frac{-\Delta E_{i}}{RT}\right)}{\sum_{i}\exp\left(\frac{-\Delta E_{i}}{RT}\right)}\times 100$$

in which, Δ*E*_*i*_ denotes the difference between the value of BE^CP^ for complex *i* (dimer or trimer *i* in the present study) and the most stable dimer or the most stable trimer with the lowest (most negative) BE^CP^ value at ambient temperature (298.15 K). In a 1:1 mixture of **M-1** and **M-2**, the % *P* index indicates that **Dim-TB** constitutes 61.19% of the mixture, **Dim-XB** 38.15%, and **Dim-HB** a negligible 0.66%. These results, consistent with the binding energy (BE^CP^) values, confirm that **Dim-TB** is the most stable and thus the most prevalent dimer complex. Similarly, for the trimers, **Trim-THB**, being the most stable, also shows the highest abundance. In the case of a 1:3 mixture of **M-1** and **M-2**, **Tet-THXB** is the only complex present, accounting for 100% of the mixture. It is important to note that despite a small difference of just 0.28 kcal·mol^–1^ in the BE^CP^ value between **Dim-TB** and **Dim-XB**, the % *P* for **Dim-TB** is nearly double that of **Dim-XB**. This is due to the exponential nature of Boltzmann’s distribution, where even a 1 kcal·mol^–1^ increase in stability can change the percentage by a factor of 5.4 at ambient temperature.

In the study of NCIs, the total interaction energy in molecular assemblies should not be regarded solely as the sum of pairwise (two-body) interactions, as this approach neglects higher-order contributions (e.g., three-body, four-body interactions). In certain cases, these higher-order interactions can have a significant impact on the overall stability and properties of the system. Through “many-body” interaction analysis,^31^ we can gain insight into how the interaction energy (IE^CP^) of a given complex is distributed among two-body, three-body, and four-body contributions. Table 2, in addition to the values of IE^CP^, summarizes the two-, three-, and four-body contributions for the complexes studied, automatically generated by the PSI4 software during the “focal point” computations.

**Table 2:** Computed Value^a^ of Counterpoise-Corrected Interaction Energy, IE^CP^, Together with Its Decomposition Into the CP-Corrected Two-, Three-, and Four-Body Contributions, in kcal·mol^–1^, for the Dimers, Trimers, and Tetramer Investigated

complex	IE^CP^	two-body contribution	three-body contribution	four-body contribution
**Dim-TB**	–11.31	–11.31		
**Dim-HB**	–1.83	–1.83		
**Dim-XB**	–4.29	–4.29		
**Trim-THB**	–18.88	–18.02	–0.86	
**Trim-TXB**	–11.08	–11.64	0.56	
**Trim-HXB**	–7.13	–6.39	–0.74	
**Tet-THXB**	–19.53	–18.82	–0.73	0.02

a All terms are extrapolated toward CBS limit employing focal point approach.

As expected, the IE^CP^ for dimers is entirely distributed among two-body interactions. For trimers, the energy is predominantly due to two-body interactions, with a negligible contribution from three-body interactions. Notably, in **Trim-THB** and **Trim-HXB**, the three-body contribution is constructive (negative), while in **Trim-TXB**, it is positive, indicating a destabilizing effect. In the case of **Tet-THXB**, the two-body interaction is the main contributor, with minor constructive three-body and negligible four-body interactions.

It is important to note that the values reported in Table 1 are based on the assumption that dimers, trimers, and the tetramer are directly formed through encounters between separate monomers via bimolecular (**M-1** + **M-2**), trimolecular (**M-1** + **2M-2**), and tetra-molecular (**M-1** + **3M-2**) reactions. In other words, the BE^CP^ and % *P* values in Table 1 reflect the energy contributions of each complex relative to the completely separated monomers. According to basic kinetics principles, as the molecularity of a reaction increases, the likelihood of “effective encounters” and, thus, the probability of the reaction occurring decreases. Therefore, while the formation of dimers is feasible, the direct formation of trimers and the tetramer from separate **M-1** and **M-2** molecules, requiring tri- and tetra-molecular reactions, is kinetically very unfavorable. Instead, these complexes likely form through a *stepwise* mechanism. In this mechanism, **Dim-TB**, the most stable dimer, is initially formed through the encounter of **M-1** and **M-2**. As depicted in Scheme 2, **Dim-TB** can then convert into **Trim-THB** or **Trim-TXB**. However, based on Table 1, **Trim-THB** (*P* = 82.27%) is significantly more stable than **Trim-TXB** (*P* = 8.72%). Therefore, the interaction of the already formed **Dim-TB** with another **M-2** molecule is more likely to form **Trim-THB**. Finally, **Trim-THB**, upon encountering another **M-2** molecule, forms the only tetramer, **Tet-THXB**. Thus, a reasonable mechanism consisting of three elementary second-order reactions may be proposed as

(i) **M-1** + **M-2** → **Dim-TB**
(ii) **Dim-TB** + **M-2** → **Trim-THB**
(iii) **Trim-THB** + **M-2** → **Tet-THXB**

To provide further insight into the PES of the complexation process, Table 3 summarizes the energetic descriptors for each step in the proposed mechanism mentioned above, similar to those provided in Table 1. The data presented in Table 3 illustrates how the composition of the reaction mixture evolves when monomer **M-2** is gradually added to monomer **M-1**, drop by drop, in an experimental setup. Initially, the encounter between one molecule of **M-1** and one molecule of **M-2** forms **Dim-TB**, reducing the PES by 4.44 kcal·mol^–1^ in the first step of the proposed mechanism. Next, the already-formed **Dim-TB** interacts with an additional **M-2** molecule, resulting in the formation of **Trim-THB**, which is 4.05 kcal·mol^–1^ lower in energy than the separate **Dim-TB** and **M-2** in the second step.

**Table 3:** Computed Energetics Descriptors, in kcal·mol^–1^, for Each of Elementary Reactions Converting Separate **M-1** and **M-2** into **Tet-THXB** along a Proposed Stepwise Mechanism

complexation process	IE^raw^	*E*_BSSE_	IE^CP^	Δ*E*_M‑1_^def^	Δ*E*_M‑2_^def^	Δ*E*_tot_^def^	BE^raw^	BE^CP^
**M-1** + **M-2** → **Dim-TB**	–14.04	2.73	–11.31	6.80	0.07	6.87	–7.17	–4.44
**Dim-TB** + **M-2** → **Trim-THB**	–5.54	1.18	–4.36	0.29	0.02	0.31	–5.23	–4.05
**Trim-THB** + **M-2** → **Tet-THXB**	–3.77	0.48	–3.29	0.21	0.00	0.21	–3.56	–3.08

Finally, the encounter between **Trim-THB** and another **M-2** molecule produces **Tet-THXB**, lowering the energy by 3.08 kcal·mol^–1^ in the last step. Based on the values in Table 3, the PES for the exothermic formation of the studied complexes is depicted in Scheme 3. This scheme highlights that the Ge···N TB, O···H HB, and Cl···N XB form sequentially as the first, second, and third NCIs, leading to the formation of **Dim-TB**, **Trim-THB**, and **Tet-THXB**, respectively.

**Scheme 3:**
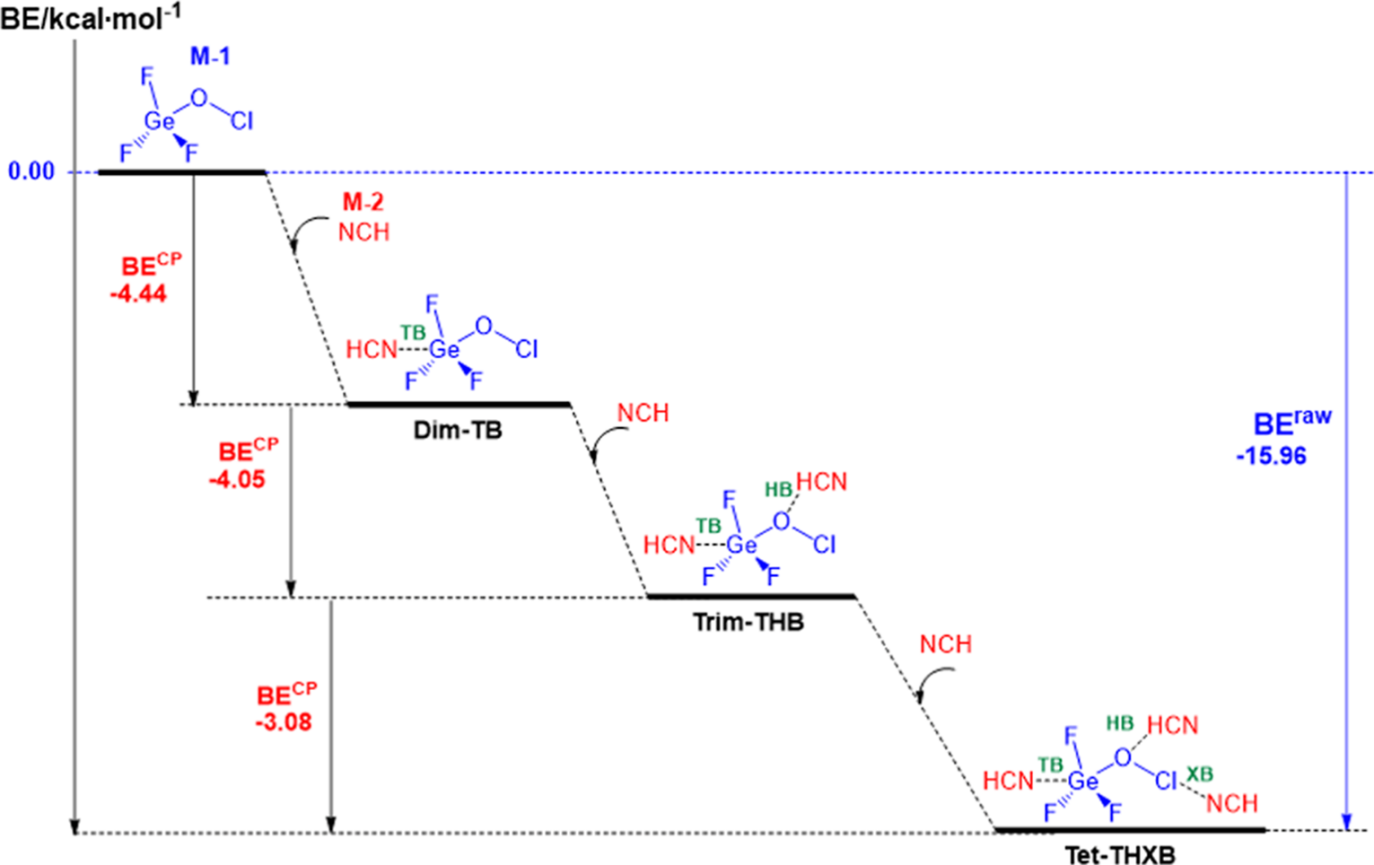
PES Associated with the Gradually Addition of Monomer **M-2** to a Great Amount of Monomer **M-1** Representing How the Dyad Complex, **Dim-TB**, Triad Complex **Trim-THB**, and the Tetrad Complex **Tet-THXB** Could Be Formed in *Real* along a *Stepwise* Mechanism Comprising of Three Second-Order Elementary Reactions^a^

a While the value of BE^CP^ is given in red color for any step, the value of overall BE^raw^ is given in blue color.

It is important to note that, in calculating the overall BE^CP^, we cannot simply sum the BE^CP^ values for each step in Scheme 3 (the red values shown are taken from Table 3). This is because the BSSE correction (*E*_BSSE_) is dependent on the specific basis functions used, and therefore differs for each step. Consequently, as shown by a blue color in Scheme 3, we can only present the overall BE^raw^ value for the entire proposed mechanism, which is −15.96 kcal·mol^–1^. Indeed

5 $$M‐1\overset{M‐2}{\to }\mathrm{Dim}‐\text{TB}\overset{M‐2}{\to }Trim‐THB\overset{M‐2}{\to }Tet‐THXB,{\text{BE}}_{\text{overall}}^{\text{raw}}=-15.96\;{\text{kcal}\cdot \text{mol}}^{-1}$$

When more than one interaction exists in an assembly of molecules, a very important concept namely cooperativity is deserving attention. The cooperative phenomenon makes sense when, at least, three monomers interact with each other in a given ternary complex (trimer). Here, we are interested in exploring how NCIs in **Trim-THB** and **Tet-THXB**, as the main contributing complexes resulting from encounters between **M-1** and **M-2**, mutually affect each other. To this end, a quantity namely counterpoise-corrected cooperative energy, *E*_coop_^CP^, should be computed whose sign and magnitude characterize, respectively, type and strength of interplay between NCIs of interest (see introduction). The value of *E*_coop_^CP^ for **Trim-THB** could be calculated using eq 6 as follows^64,65^

6 $$E_{\text{coop}}^{\text{CP}}={\text{IE}}^{\text{CP}}{\left(\text{Trim}‐\text{THB}\right)}_{\text{Trim}‐\text{THB}}-\left[{\text{IE}}^{\text{CP}}{\left(\mathrm{Dim}‐\text{TB}\right)}_{\mathrm{Dim}‐\text{TB}}+{\text{IE}}^{\text{CP}}{\left(\mathrm{Dim}‐\text{HB}\right)}_{\mathrm{Dim}‐\text{HB}}+{\text{IE}}^{\text{CP}}{\left(B\cdot \cdot \cdot C\right)}_{\text{Trim}‐\text{THB}}\right]$$

where, IE^CP^(Trim-THB)_Trim‑THB_ is the CP-corrected interaction energy of the fully optimized **Trim-THB**, IE^CP^(Dim-TB)_Dim‑TB_ and IE^CP^(Dim-HB)_Dim‑HB_ stand for the CP-corrected interaction energy of separately fully optimized **Dim-TB** and **Dim-HB**, respectively, and IE^CP^(B···C)_Trim‑THB_ denotes the CP-corrected energy for the interaction between B and C (see Scheme 4 in which A, B, C, and D are specified), frozen at the fully optimized structure of **Trim-THB**. Similarly, the value of *E*_coop_^CP^ for **Tet-THXB** may be evaluated employing eq 7 given as^64,65^

7 $$E_{\text{coop}}^{\text{CP}}={\text{IE}}^{\text{CP}}{\left(\text{Tet}‐\text{THXB}\right)}_{\text{Tet}‐\text{THXB}}-\left[{\text{IE}}^{\text{CP}}{\left(\mathrm{Dim}‐\text{TB}\right)}_{\mathrm{Dim}‐\text{TB}}+{\text{IE}}^{\text{CP}}{\left(\mathrm{Dim}‐\text{HB}\right)}_{\mathrm{Dim}‐\text{HB}}+{\text{IE}}^{\text{CP}}{\left(\mathrm{Dim}‐\text{XB}\right)}_{\mathrm{Dim}‐\text{XB}}+{\text{IE}}^{\text{CP}}{\left(B\cdot \cdot \cdot D\right)}_{\text{Tet}‐\text{THXB}}+{\text{IE}}^{\text{CP}}{\left(B\cdot \cdot \cdot C\right)}_{\text{Tet}‐\text{THXB}}+{\text{IE}}^{\text{CP}}{\left(C\cdot \cdot \cdot D\right)}_{\text{Tet}‐\text{THXB}}\right]$$

In eq 7, IE^CP^(Tet-THXB)_Tet‑THXB_ is the CP-corrected interaction energy of the fully optimized **Tet-THXB**, IE^CP^(Dim-TB)_Dim‑TB_, IE^CP^(Dim-HB)_Dim‑HB_, and IE^CP^(Dim-XB)_Dim‑XB_ reveal the CP-corrected interaction energy of separately fully optimized **Dim-TB**, **Dim-HB**, and **Dim-XB**, respectively. IE^CP^(B···D)_Tet‑THXB_, IE^CP^(B···C)_Tet‑THXB_, and IE^CP^(C···D)_Tet‑THXB_ stand for the CP-corrected energy for the interaction between B and D, between B and C and, between C and D, respectively, all frozen in the fully optimized structure of **Tet-THXB**, as shown in Scheme 4.

**Scheme 4:**
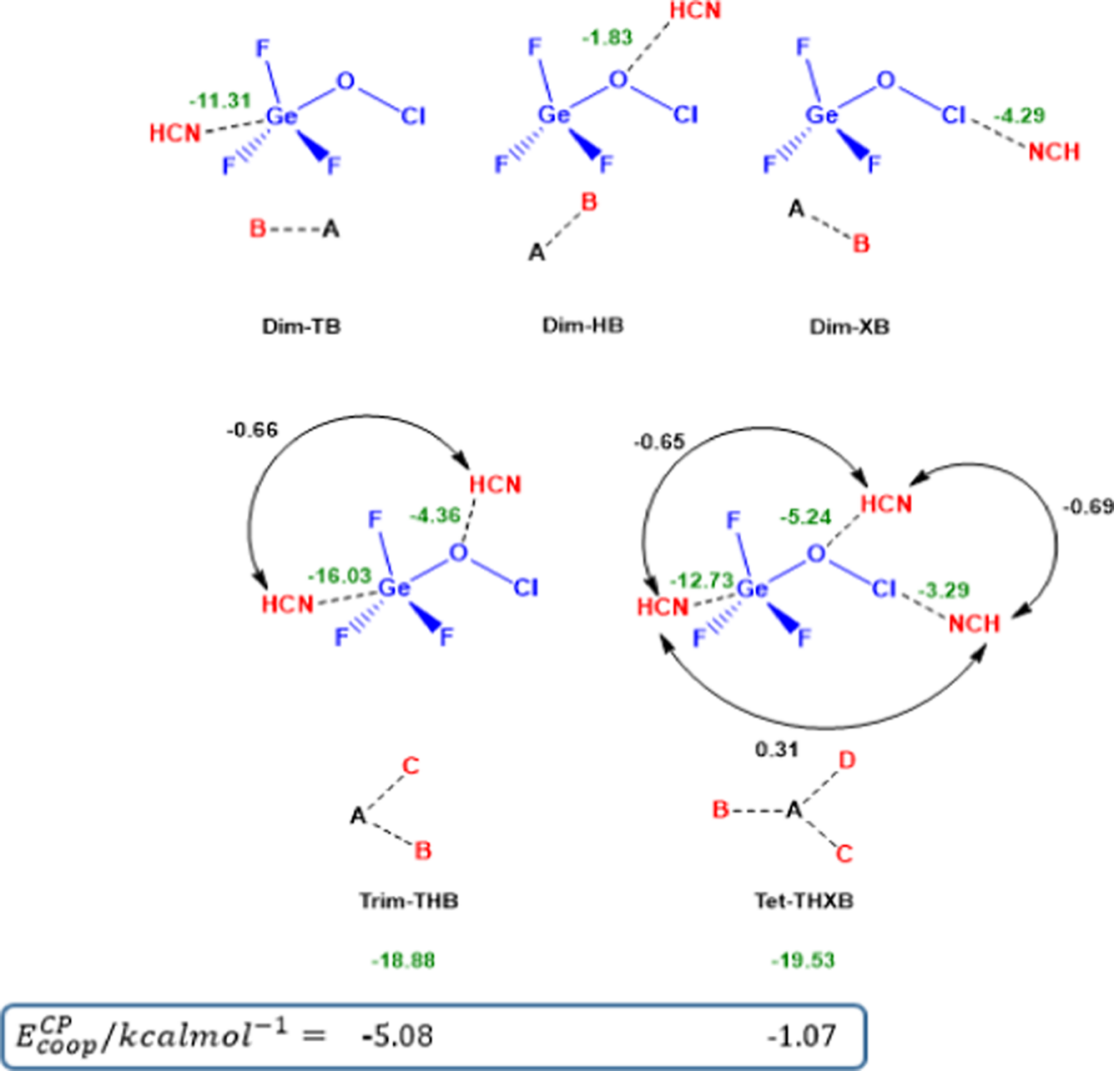
Geometry Structure of **Dim-TB**, **Dim-HB**, **Dim-XB**, **Trim-THB**, and **Tet-THXB** Together with the Values of IE^CP^ Corresponding to Different Interactions Required to Evaluate the Value of *E*_coop_^CP^ in **Trim-THB**, and **Tet-THXB**. See the Text for the Meaning of Numerical Values in Different Colors

The geometric structures and calculated IE^CP^ values for the complexes, which are key to determining the cooperative energy *E*_coop_^CP^ in **Trim-THB** and **Tet-THXB**, are shown in Scheme 4. The IE^CP^ values for the fully optimized dimers—**Dim-TB** (−11.31 kcal·mol^–1^), **Dim-HB** (−1.83 kcal·mol^–1^), and **Dim-XB** (−4.29 kcal·mol^–1^)—are associated with the respective TB, HB, and XB interactions. However, the IE^CP^ value in **Trim-THB** (−18.88 kcal·mol^–1^) and **Tet-THXB** (−19.53 kcal·mol^–1^), which involve multiple interactions, is provided for the entire complex. In **Trim-THB**, the IE^CP^ for each interaction is calculated by treating the complex as two interacting monomers. For example, the Ge···N TB interaction value of −16.03 kcal·mol^–1^ is obtained by considering **Trim-THB** as **Dim-HB** interacting with **M-2**, while the O···H HB value of −4.36 kcal·mol^–1^ is derived by treating it as **Dim-TB** interacting with **M-2**. Similarly, for **Tet-THXB**, the IE^CP^ values are −12.73 kcal·mol^–1^ for TB, −5.24 kcal·mol^–1^ for HB, and −3.29 kcal·mol^–1^ for XB, calculated by considering interactions between **Trim-HXB**, **Trim-TXB**, or **Trim-THB** with **M-2**. These values reflect the mutual influence of other interactions within the complex. The black values in Scheme 4 denote the IE^CP^ for interactions newly formed in the complex, corresponding to the last term and three last terms on the right-hand side of eqs 6 and 7, respectively. Based on the data in Scheme 4 and eqs 6 and 7, the *E*_coop_^CP^ for **Trim-THB** and **Tet-THXB** is calculated to be −5.08 and −1.07 kcal·mol^–1^, respectively.

A comparison between the IE^CP^ for the Ge···N TB (−11.31 kcal·mol^–1^ in **Dim-TB**) and O···H HB (−1.83 kcal·mol^–1^ in **Dim-HB**) with the corresponding values in **Trim-THB** (−16.03 and −4.36 kcal·mol^–1^) clearly shows that both interactions are strengthened in **Trim-THB**. This cooperativity is evident from the significantly negative *E*_coop_^CP^ value of −5.08 kcal·mol^–1^. As shown in Scheme 4, the difference in IE^CP^ between the Ge···N TB interaction in **Dim-TB** and **Trim-THB** is nearly twice that of the O···H HB interaction, suggesting that in **Trim-THB**, the HB exerts a more pronounced influence on the TB than vice versa. A closer look at Scheme 4 reveals that the IE^CP^ for the Ge···N TB interaction shifts from −11.31 kcal·mol^–1^ in **Dim-TB** to −12.73 kcal·mol^–1^ in **Tet-THXB**, indicating that the O···H HB and Cl···N XB interactions reinforce the Ge···N TB in **Tet-THXB**. Similarly, the O···H HB interaction becomes significantly stronger, with its IE^CP^ changing from −1.83 kcal·mol^–1^ in **Dim-HB** to −5.24 kcal·mol^–1^ in **Tet-THXB**, showing the positive mutual influence of the Ge···N TB and Cl···N XB interactions. On the other hand, the Cl···N XB interaction weakens in the presence of the Ge···N TB and O···H HB interactions, as evidenced by a less negative IE^CP^ value in **Tet-THXB** (−3.29 kcal·mol^–1^) compared to **Dim-XB** (−4.29 kcal·mol^–1^). Thus, in **Tet-THXB**, the Ge···N TB and O···H HB interactions become stronger, while the Cl···N XB interaction weakens. The overall effect of these interactions results in weak cooperativity, characterized by a slightly negative *E*_coop_^CP^ of −1.07 kcal·mol^–1^. Furthermore, the IE^CP^ values for each interaction in the absence of the others were estimated to be −12.34 kcal·mol^–1^ for Ge···N TB, −2.46 kcal·mol^–1^ for O···H HB, and −2.97 kcal·mol^–1^ for Cl···N XB in the fully optimized **Tet-THXB** structure. This demonstrates that, compared to the corresponding dimers, the TB and HB interactions strengthen, while the XB interaction weakens when considered independently in **Tet-THXB**.

At this point, it should be mentioned that these results could be predicted qualitatively by simply analyzing which pairs of bonds make the central molecule a double electron donor or acceptor, and in which does the central unit serve in both capacities simultaneously. It is commonly accepted that the first mode leads to negative cooperativity, while synergy occurs for the latter.

### 3.4 Geometrical Parameters, QTAIM Topological Descriptors, IGM and IBSI Analysis

Geometrical-based descriptors are the simplest and most accessible tools for detecting and confirming the presence of NCIs. Lu, in the Multiwfn software manual, proposed a practical and geometrical-based index known as mutual penetration distance (M.P.D.), which is defined for a given X···Y interaction as follows^66^

8 $$M.P.D.={\left(n.b.r\right)}_{X}+{\left(n.b.r\right)}_{Y}-d\left(X\cdot \cdot \cdot Y\right)$$

where, (n.b.r)_X_ and (n.b.r)_Y_ represent the nonbonded radius of atoms X and Y involved in the X···Y NCI, and *d*(X···Y) is the equilibrium distance between X and Y. According to QTAIM, the nonbonded radius of an atom, also referred to as the van der Waals (vdW) radius, is the shortest distance between its nucleus and the ρ = 0.001 au surface that surrounds the complex. Typically, the equilibrium distance between two atoms in an NCI is shorter than the sum of their vdW radii. Hence, by definition, M.P.D. should always be positive when an NCI is present. As the NCI strengthens, the electron cloud penetration deepens, resulting in a higher M.P.D. value. The TB, HB, and XB distances, along with their corresponding M.P.D. values for the studied complexes, are presented in Table 4. Focusing on the most relevant species after **Dim-TB** formation—**Trim-THB** and **Tet-THXB**—Table 4 shows that the Ge1···N5 TB distance (2.293 Å) and O2···H4 HB distance (2.059 Å) in **Trim-THB** are shorter than the corresponding distances in **Dim-TB** (2.397 Å) and **Dim-HB** (2.163 Å), resulting in larger M.P.D. values in **Trim-THB**. This supports this finding that TB and HB interactions reinforce each other through cooperativity. Further inspection reveals that in **Tet-THXB**, the Ge1···N5 TB (2.359 Å) and O2···H4 HB (2.043 Å) distances are also shorter than those in the corresponding dimers. However, the Cl3···N5 XB distance in **Tet-THXB** (2.756 Å) is slightly longer than in **Dim-XB** (2.700 Å). These differences result in higher M.P.D. values for TB and HB interactions and a lower M.P.D. value for XB in **Tet-THXB**, aligning with the energetics data and further confirming the cooperativity in these systems.

**Table 4:** Value of TB-Distance, *d*(Ge1···N5), HB-Distance, *d*(O2···H4), and XB-Distance, *d*(Cl3···N5), Together with the Value of Mutual Penetration Distance (M.P.D.), in Å, in the MP2/aug-cc-pVDZ Fully Optimized Geometry of Dimers, Trimers, and the Only Tetramer Formed between **M-1** and **M-2** (Atom Numbering Is Given in Scheme 2)

complex	TB	HB	XB	M.P.D.		
	*d*(Ge1···N5)	*d*(O2···H4)	*d*(Cl3···N5)	(Si1···N5)TB	(O2···H4)HB	(Cl3···N5)XB
**Dim-TB**	2.397			1.336		
**Dim-HB**		2.163			0.917	
**Dim-XB**			2.700			1.204
**Trim-THB**	2.293	2.059		1.444	1.076	
**Trim-TXB**	2.524		2.798	1.203		1.112
**Trim-HXB**		2.127	2.639		0.975	1.271
**Tet-THXB**	2.359	2.043	2.756	1.375	1.104	1.154

In alignment with the findings from the interaction energy IE^CP^ values in Scheme 4, the geometrical descriptors further confirm that the tetrel (TB) and hydrogen bond (HB) interactions become stronger, while the halogen bond (XB) weakens in **Tet-THXB**.

Within the framework of QTAIM theory, the bond critical point (BCP) is the key point along the interaction path between two atoms. Using the MP2/aug-cc-pVDZ method, several QTAIM topological descriptors were calculated at the BCPs of the N···Ge, Cl···N, and O···H interactions in the studied complexes. These descriptors include charge density (ρ), Laplacian of charge density (∇^2^ρ), energy density (*H*), and the ratio of absolute potential energy density to Lagrangian kinetic energy ( $\frac{\left|V\right|}{G}$ ). The values, along with the QTAIM molecular graph for each complex, are shown in Figure 3. Generally, a positive ∇^2^ρ at the BCP is associated with noncovalent interactions, while a negative value indicates covalent interactions.^65^ However, relying solely on ∇^2^ρ can lead to misinterpretation; for example, the covalent C–O bond in CO displays a positive ∇^2^ρ at its BCP.^67^ Espinosa^68^ suggested that the |*V*|/*G* ratio is a more reliable indicator, where values less than 1 indicate a purely noncovalent interaction (e.g., HB, XB, TB), values between 1 and 2 suggest a bond with partial covalent character, and values above 2 represent a fully covalent interaction.

**Figure 3:**
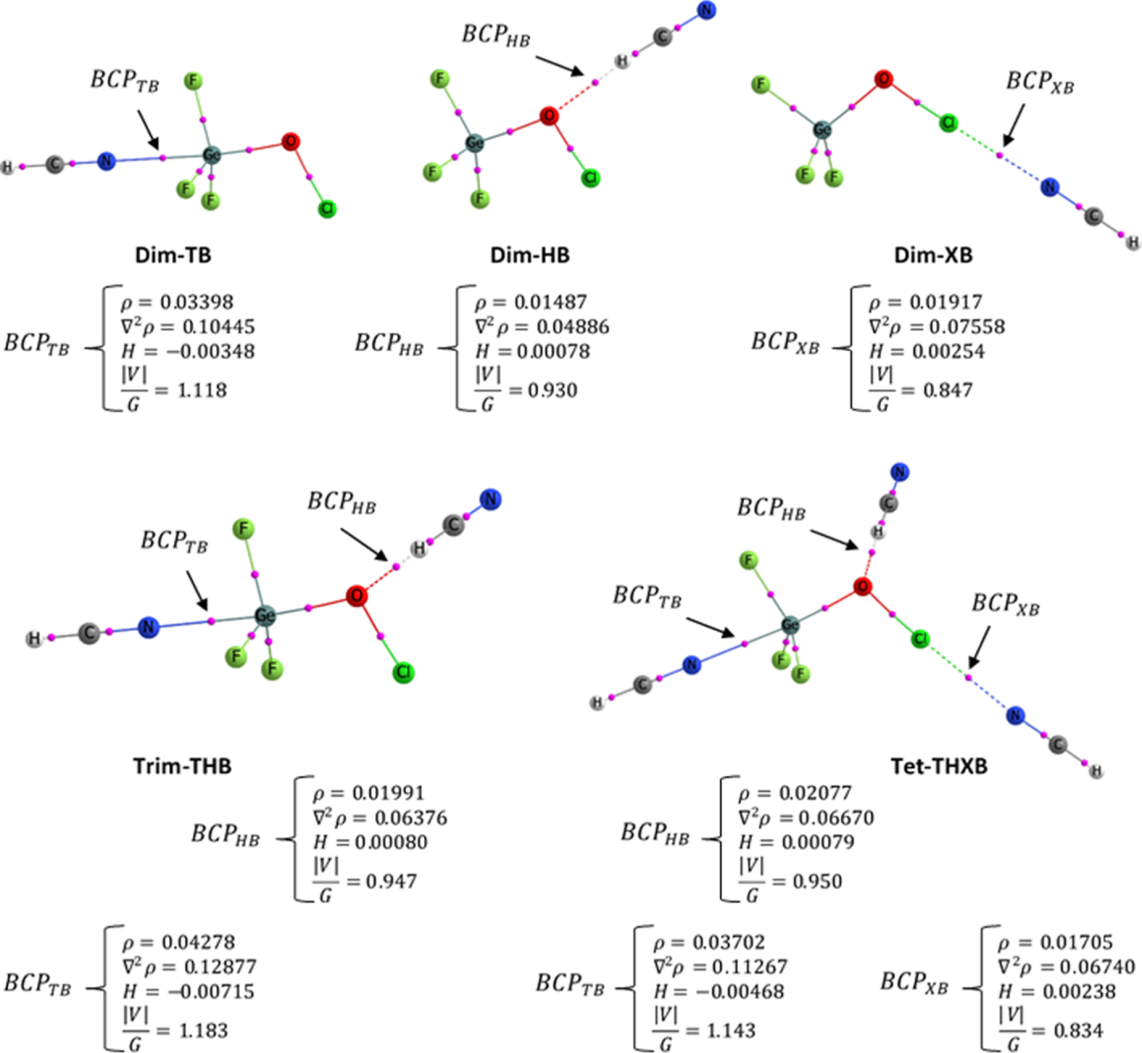
MP2/aug-cc-pVDZ QTAIM molecular graphs of the assemblies whose consideration is necessary to determine the reaction mixture’s composition. The small pink circles characterize BCP positions while the target BCPs are evidently specified and referred to by a black arrow for which the value of ρ, ∇^2^ρ, *H*, in au, and |*V*|/*G*, dimensionless, is given as well.

The strength of an interaction is reflected in the ρ value at the BCP: higher ρ values correspond to stronger interactions. A ρ value less than 0.1 au indicates a noncovalent interaction, while values above 0.2 au indicate covalent interactions. Values between these limits suggest an NCI with partial covalent character.^69^

Additionally, the energy density (*H* = *G* + *V*) offers insight into the nature of the interaction. Positive *H* values indicate electrostatic interactions, while negative values suggest covalent bonding, with increasingly negative values indicating stronger covalent character.^70^ As shown in Figure 3, all the studied complexes exhibit ρ values less than 0.1 au and positive ∇^2^ρ values, confirming the primarily electrostatic nature of the TB, HB, and XB NCIs. However, in the Ge···N TB interaction, *H* is slightly negative, and |*V*|/*G* exceeds 1, indicating a minor covalent contribution. Therefore, a slight covalent character can be inferred for the Ge···N TB in these complexes. The cooperativity of NCIs in **Trim-THB** and **Tet-THXB** was thoroughly demonstrated in the previous sections. In **Trim-THB**, the value of ρ at the BCP of the Ge···N (0.04278 au) and O···H (0.01991 au) interactions is greater than that of the corresponding Ge···N (0.03398 au) in **Dim-TB** and O···H (0.01487 au) in **Dim-HB**. This indicates that both interactions not only strengthen individually but also reinforce each other when present together in **Trim-THB**, in line with the results from other analyses.

Furthermore, the ρ values at the BCPs of the Ge···N TB (0.03398 au), O···H HB (0.01487 au), and Cl···N XB (0.01917 au) interactions in **Dim-TB**, **Dim-HB**, and **Dim-XB** change to 0.03702, 0.02077, and 0.01705 au, respectively, in **Tet-THXB**. Consequently, from the dimers to **Tet-THXB**, the TB and HB interactions strengthen by 8.95% and 39.70%, while the XB weakens by 11.06%. In other words, in **Tet-THXB**, the HB and XB interactions reinforce the TB interaction (cooperative effect), and TB and XB reinforce the HB interaction (cooperative effect). However, the TB and HB interactions diminish the XB interaction (diminutive effect). Altogether, these effects result in a modest cooperativity in **Tet-THXB**, with a small cooperative energy *E*_coop_^CP^ = −1.07 kcal·mol^–1^ (Section 3.3). This model clearly illustrates how interactions can be modulated by the presence of others.

As shown in Figure 4, a linear regression of IE^CP^ values (given in Scheme 4) against the corresponding ρ values at the BCP (given in Figure 3) results in an excellent correlation (*R* ^2^ = 0.9983), indicating that over 99% of the changes in IE^CP^ can be explained by changes in ρ values. This correlation provides a highly reliable method for predicting IE^CP^ values, which are computationally expensive to calculate, by using the fast and easily computable ρ values at the BCPs of the NCIs.

**Figure 4:**
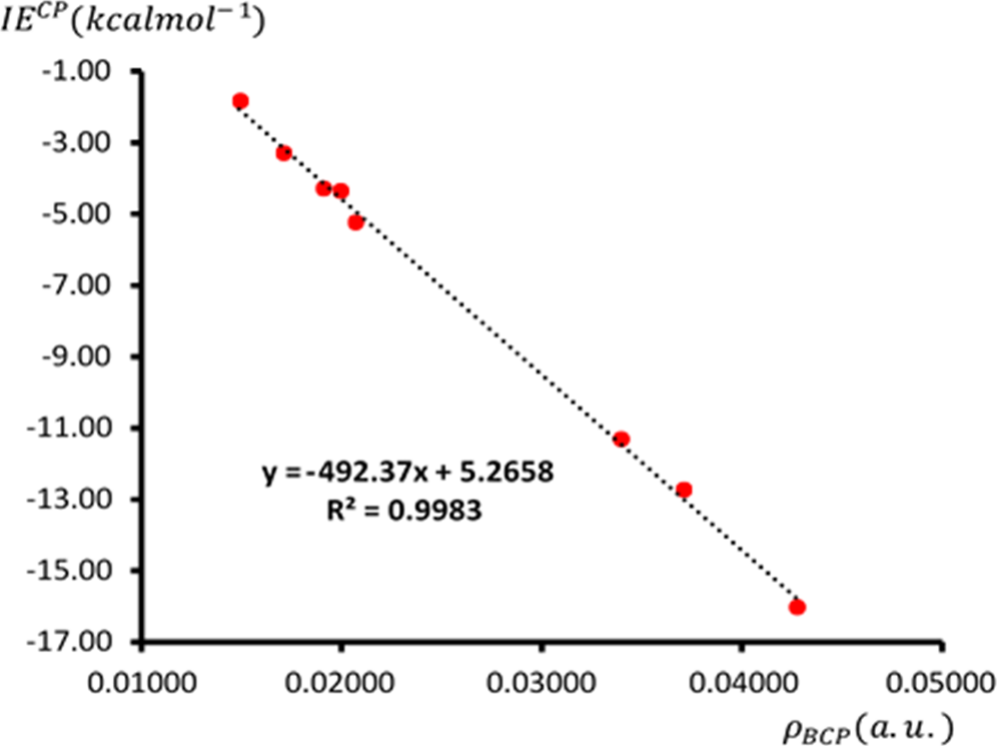
2-D linear regression plot of CBS-extrapolated values of IE^CP^ associated with Ge···N TB, O···H HB, and Cl···N XB NCIs in **Dim-TB**, **Dim-HB**, **Dim-XB**, **Trim-THB**, and **Tet-THXB** versus MP2/aug-cc-pVDZ values of ρ at the corresponding BCPs.

Lefebvre et al. recently introduced an electron density-based descriptor called the Independent Gradient Model (IGM), which allows for accurate and distinct characterization of NCIs and covalent bonds.^53^ In the IGM approach, a 2D plot of intermolecular contributions to the gradient of electron density, denoted as δ*g* ^inter^, versus sign (λ_2_)­ρ (where λ_2_ is the second largest eigenvalue of the Hessian matrix of ρ), leads to pop up clear spikes at negative but small *X*-axis values, indicating the presence of NCIs in a molecular system.^71^ The *X*-axis position of a spike corresponds to the electron density ρ and thus reflects the strength of the interaction, while the *Y*-axis height of the spike (δ*g* ^inter^) has been shown to correlate with IE values.^55,71^ Additionally, 3D IGM isosurfaces colored blue, green, and red provide intuitive visual cues about the strength of NCIs: blue indicates strong attraction, green denotes weakly attractive or destructive vdW interactions, and red suggests strong repulsion. A useful numerical descriptor introduced by Lefebvre, the “δ*g* peak height”, helps classify interactions: weak NCIs rarely exceed 0.1 au, vdW interactions range from 0.02 to 0.03 au, and hydrogen bonds (HB) can reach up to 0.1 au pure covalent bonds show δ*g* peak heights between 0.2 and 1.0 au, while metal coordination interactions fall between 0.1 and 0.6 au.^55^

Another valuable indicator within the IGM framework is the intrinsic bond strength index (IBSI), which quantifies and compares the strength of interactions.^54^ As the strength of an interaction increases, so does the IBSI value, which is dimensionless and defined by

9 $$\text{IBSI}=\frac{\left(\frac{1}{d^{2}}\right)\int \delta g^{\text{pair}}\;dr}{\left(\frac{1}{d_{H_{2}}^{2}}\right)\int \delta g^{H_{2}}\;dr}$$

where *d* represents the distance between the two atoms involved in the interaction. The integral in the numerator corresponds to δ*g* ^pair^, the atom pair gradient contribution between the two interacting atoms. In the denominator, $d_{H_{2}}^{2}$ and the integral represent the bond length and atom pair δ*g* index for the H_2_ molecule in its equilibrium geometry. The IBSI value has been shown to positively correlate with the strength of the interaction.^54,55,71^ It is important to note that since both the numerator and denominator are dimensionally the same, IBSI is a dimensionless quantity. Based on a large data set of 235 species and 677 atom pairs, Lefebvre et al.^53^ developed a practical numerical scale for IBSI, which classifies interactions as follows: (i) for NCIs, IBSI ≤ 0.15, (ii) for transition metal coordination, 0.15 ≤ IBSI ≤ 0.60, and (iii) for covalent interactions, 0.15 ≤ IBSI ≤ 4.00.

Using the MP2/aug-cc-pVDZ monodeterminantal wave function, we performed IGM analysis to investigate the NCIs in the studied complexes. The corresponding 2D plots, 3D isosurfaces, and IBSI values for the atom pairs involved are shown in Figure 5.

**Figure 5:**
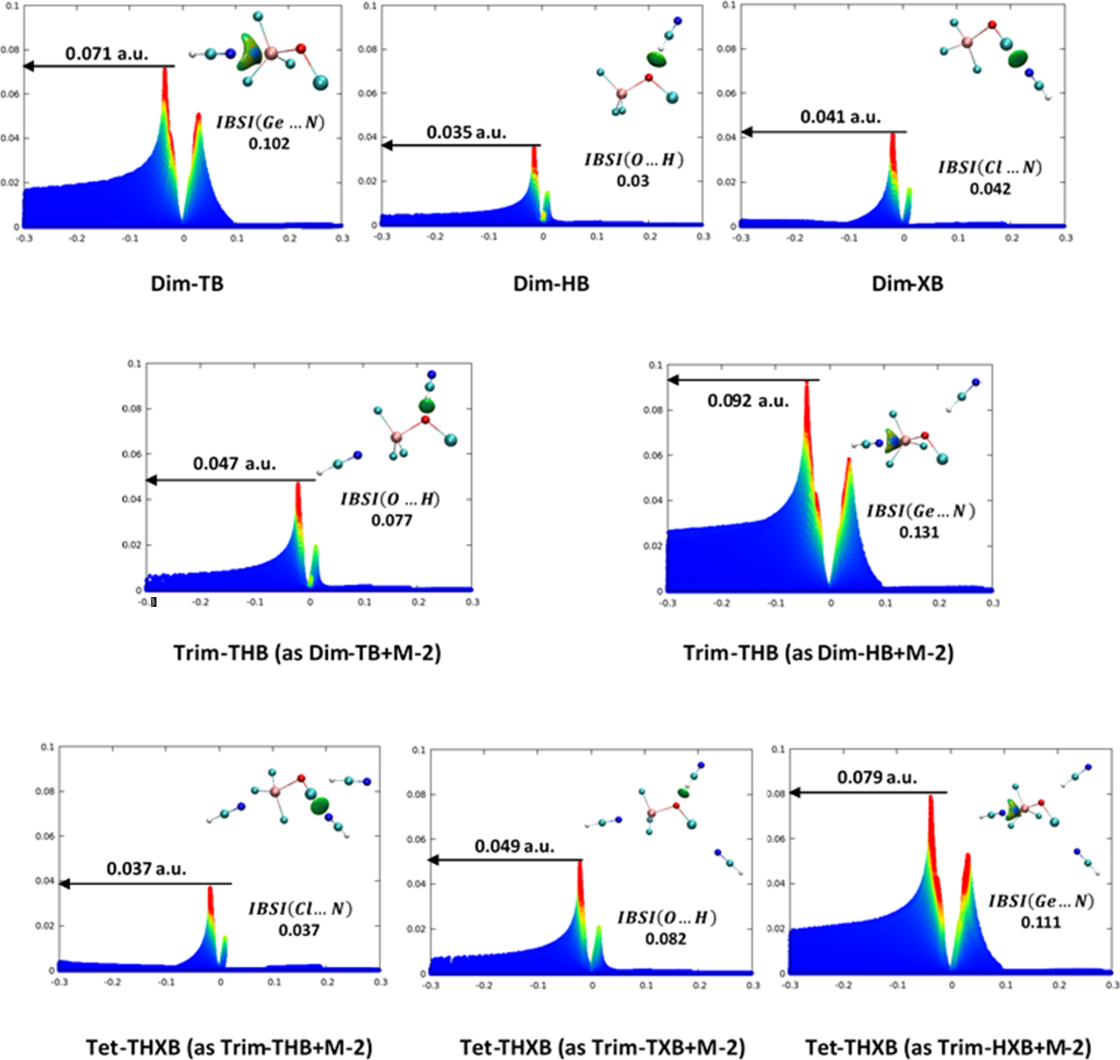
2-D plot of the values of δ*g* ^inter^ (*Y*-axis) against values of sign­(λ_2_)­ρ (*X*-axis) colored between −0.08 to 0.08 au for various NCIs in the complexes considered. The 3-D isosurface of IGM (with an electron density isovalue of 0.01 au) for the specific NCI considered is also portrayed in the top right corner. The strength of the target NCI is characterized with the δ*g* ^inter^ peak height value given over a black horizontal arrow, the value of corresponding IBSI at the bottom right corner and, the type and intensity of the color at the disk center of the IGM isosurface (see text for more interpretations).

The δ*g* ^inter^ peak heights and IBSI values for the Ge···N TB in **Dim-TB**, O···H HB in **Dim-HB**, and Cl···N XB in **Dim-XB** are 0.071 au (IBSI 0.102), 0.035 au (IBSI 0.03), and 0.041 au (IBSI 0.042), respectively. Notably, the Ge···N TB interaction in **Dim-TB** is depicted with a 3D IGM disk with a blue region at its center, indicating strong attraction, while the HB and XB interactions in **Dim-HB** and **Dim-XB** show green-colored disks, with the XB complex displaying a slightly larger, more pronounced green region. Consequently, the values of the δ*g* ^inter^ peak height and IBSI, along with the type and intensity of color in the IGM 3D disk, indicate that the strength of the NCIs follows the order: TB in **Dim-TB** > XB in **Dim-XB** > HB in **Dim-HB**, consistent with previous analyses. Moreover, the δ*g* ^inter^ peak height and IBSI values are 0.047 au and 0.077 for the O···H HB, and 0.092 au and 0.131 for the Ge···N TB in **Trim-THB** (treated as **Dim-TB** + **M-2**). Comparing these values with those in **Dim-HB** and **Dim-TB**, it becomes clear that both O···H HB and Ge···N TB interactions are stronger in **Trim-THB**, and also reinforce each other, in line with the cooperativity effect observed in **Trim-THB** (see Section 3.3). A closer examination of the δ*g* ^inter^ peak height and IBSI values in Figure 5 further reveals that both the Ge···N interaction in **Dim-TB** and the O···H HB interaction in **Dim-HB** become stronger, while the Cl···N XB interaction in **Dim-XB** weakens in **Tet-THXB** compared to their respective dimers. In **Tet-THXB**, consistent with previous analyses, the TB and HB interactions reinforce each other (a cooperative effect), while the XB interaction becomes weaker due to the influence of the TB and HB interactions (a diminutive effect).

### 3.5 Energy Decomposition Analysis (EDA) of the Complexes

EDA is a highly valuable method used to break down the total interaction energy between fragments in chemical systems into distinct and physically meaningful components. This allows chemists to identify which factors play a dominant role in the interaction, offering clear insights into its nature. Several EDA methods have been developed and widely used, including NEDA,^72^ LMOEDA,^73^ GKS-EDA,^74^ Kitaura–Morokuma,^75^ ALMO-EDA,^76^ and SAPT,^56^ all of which have been extensively applied in theoretical studies.^77–80^ Among these, SAPT is particularly well-known for its accuracy and its ability to decompose interaction energies into various contributing components, providing valuable understanding of the nature of a given interaction.^81^ SAPT avoids calculating the energy of the whole system or individual monomers, making it free from BSSE issues, unlike the supermolecular (SM) approach.^81^ In SAPT, the IE between monomers is decomposed as follows^58^

10 $${\text{IE}}_{\text{SAPT}}=E_{\text{ele}}+E_{\text{exc}}+E_{\text{ind}}+E_{\text{dis}}$$

where *E*_ele_ represents electrostatic contributions, *E*_exc_ is the exchange-repulsion term, *E*_ind_ accounts for induction and charge transfer effects, and *E*_dis_ is the dispersion component arising from dynamic electron correlations.^58^

Recently, Lu introduced a new EDA method called sobEDA, and its variant sobEDAw, specifically designed for weak interaction analysis.^57^ This method, based on dispersion-corrected density functional theory (DFT), offers significant advantages such as low computational cost, ease of implementation, and high accuracy, closely matching results from high-precision CCSD­(T)/CBS and DFT-SAPT methods.^57^ SobEDAw is particularly useful for analyzing weak interactions, and when using complex basis functions (denoted as “w.CB”), the total interaction energy calculated by sobEDAw is equivalent to the IE^CP^ value obtained by the SM approach.^57^ The sobEDAw method decomposes the interaction energy as

11 $${\text{IE}}_{\text{sobEDAw}}^{\text{CP}}=\Delta E_{\text{els}}+\Delta E_{\text{xrep}}+\Delta E_{\text{orb}}+\Delta E_{\text{disp}}$$

where these terms correspond directly to the components in the SAPT method, with Δ*E*_els_ ≡ *E*_ele_, Δ*E*_xrep_ ≡ *E*_exc_, Δ*E*_orb_ ≡ *E*_ind_, and Δ*E*_disp_ ≡ *E*_dis_.

In the present study, we applied sobEDAw at the B3LYP-D3­(BJ)/6-311+G­(2d,p) w.CB level, recommended by Lu,^57^ to analyze the NCIs in MP2/aug-cc-pVDZ fully optimized complexes and to compare the results with the well-established *gold* SAPT2 + (3)­δMP2/aug-cc-pVTZ level^58^ for accuracy verification. The results are summarized in Table 5. To assess the accuracy of the SAPT and sobEDAw methods, we plotted IE_SAPT_ and IE_sobEDAw_^CP^ values against the reference IE^CP^ values from Scheme 4. As illustrated in Figure 6, both methods show an excellent correlation with reference data, yielding high *R* ^2^ values of 0.9992 for SAPT and 0.9914 for sobEDAw. Although the difference in correlation is minimal, both SAPT and sobEDAw methods effectively estimate the interaction strengths of the NCIs, providing results nearly identical to the SM reference approach. This strong correlation confirms that SAPT remains reliable in this context, with the perturbation degree low enough to justify its use.^56,58^

**Table 5:** B3LYP-D3­(BJ)/6-311+G­(2d,p) w.CB sobEDAw and SAPT2 + (3)­δMP2/aug-cc-pVTZ IE^CP^ Together with the Corresponding Components^a^ Values, in kcal·mol^–1^, and, Percent of Attractive (Instructive) Components (% Δ*E*_*X*_)­^b^ over the MP2/aug-cc-pVDZ Fully Optimized Complexes Considered

complex	IE_sobEDAw_^CP^ (IE_SAPT_)	Δ*E*_xrep_ (*E*_exc_)	Δ*E*_els_ (*E*_ele_)	Δ*E*_orb_ (*E*_ind_)	Δ*E*_disp_ (*E*_dis_)	% ΔEels (% Eele)	% ΔEorb (% Eind)	% ΔEdisp (% Edis)
**Dim-TB**	–9.74	41.31	–26.96	–15.56	–8.53	52.81	30.48	16.71
	(−10.63)	(35.37)	(−26.21)	(−10.89)	(−8.90)	56.98	23.67	19.35
**Dim-HB**	–2.13	4.08	–2.17	–0.63	–3.41	34.94	10.14	54.91
	(−1.91)	(3.61)	(−2.10)	(−1.00)	(−2.43)	37.97	18.08	43.94
**Dim-XB**	–4.66	10.38	–7.10	–3.45	–4.49	47.21	22.94	29.85
	(−4.25)	(8.99)	(−6.75)	(−2.81)	(−3.67)	51.02	21.24	27.74
^c^ **Trim-THB(1)**	–4.95	6.75	–5.61	–2.75	–3.33	47.99	23.52	28.49
	(−4.51)	(6.00)	(−5.53)	(−2.04)	(−2.95)	52.57	19.39	28.04
^d^ **Trim-THB(2)**	–14.11	52.31	–34.75	–22.31	–9.36	52.32	33.59	14.09
	(−15.16)	(44.78)	(−33.79)	(−15.97)	(−10.17)	56.38	26.65	16.97
^e^ **Tet-THXB(1)**	–3.49	8.71	–5.55	–3.18	–3.48	45.45	26.04	28.50
	(−3.25)	(7.52)	(−5.22)	(−2.14)	(−3.41)	48.47	19.87	31.66
^f^ **Tet-THXB(2)**	–5.88	7.44	–6.59	–3.11	–3.62	49.47	23.35	27.18
	(−5.38)	(6.61)	(−6.44)	(−2.32)	(−3.22)	53.76	19.37	26.88
^g^ **Tet-THXB(3)**	–10.96	45.19	–29.53	–17.76	–8.86	52.59	31.63	15.78
	(−11.99)	(38.70)	(−28.74)	(−12.54)	(−9.41)	56.70	24.74	18.56

a Components resulting from EDA which are given in eqs 10 and 11 for SAPT and sobEDAw approaches, respectively.

b In the case of sobEDAw analysis, $\%\;\Delta E_{X}=\left(\frac{X}{\Delta E_{\text{els}}+\Delta E_{\text{orb}}+\Delta E_{\text{disp}}}\right)\times 100$ where *X* stands for each of Δ*E*_els_, Δ*E*_orb_, or Δ*E*_disp_. In the case of SAPT analysis, $\%\;\Delta E_{X}=\left(\frac{X}{E_{\text{els}}+E_{\text{orb}}+E_{\text{disp}}}\right)\times 100$ where *X* stands for each of *E*_els_, *E*_orb_, or *E*_disp_.

c **Trim-THB­(1)** treated as (**Dim-TB** + **M-2**).

d **Trim-THB­(2)** treated as (**Dim-HB** + **M-2**).

e **Tet-THXB­(1)** treated as (**Trim-THB** + **M-2**).

f **Tet-THXB­(2)** treated as (**Trim-TXB** + **M-2**).

g **Tet-THXB­(3)** treated as (**Trim-HXB** + **M-2**).

**Figure 6:**
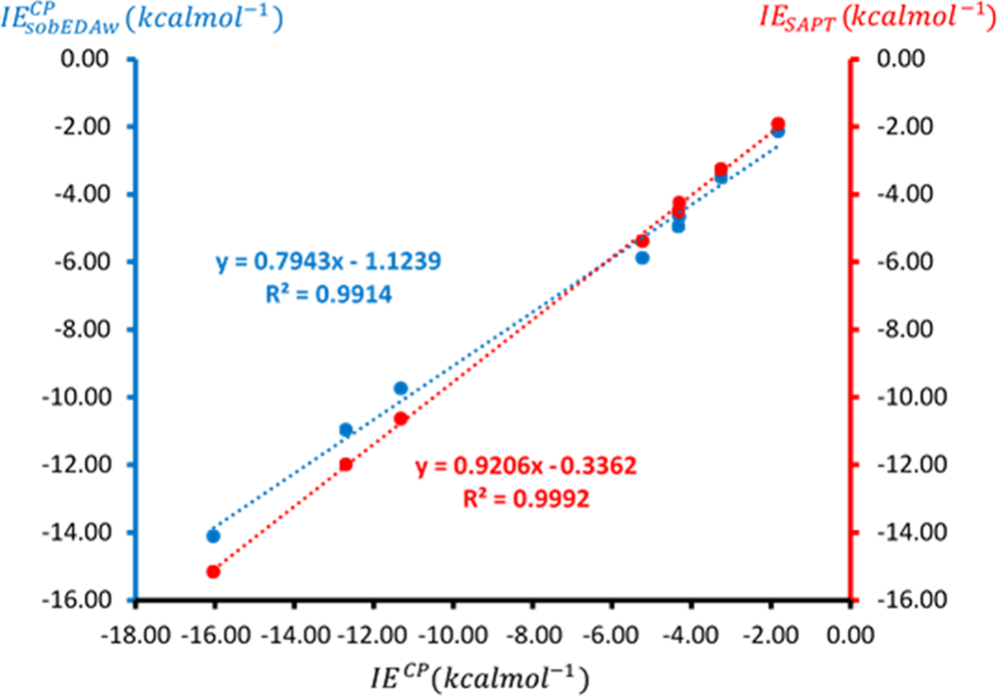
Linear correlation between values of IE_sobEDAw_^CP^ (blue color) and IE_SAPT_ (red color), taken from Table 5, with those of reference IE^CP^ associated with NCIs presented in Scheme 4.

The strong correlations illustrated in Figure 6 further confirm the excellent agreement between IE_sobEDAw_^CP^ and IE_SAPT_, highlighting the accuracy and reliability of Lu’s proposed EDA methodology. The practical significance of the estimators presented in Figure 6 lies in their ability to provide a highly accurate approximation of the CBS-extrapolated interaction energy. This approach offers a significant advantage, as calculating CBS-extrapolated values directly is highly time-consuming, whereas the sobEDAw method is both fast and easy to implement. However, it is noteworthy that the sobEDAw partition method exhibits a greater deviation from an ideal slope of 1 (0.79) compared to SAPT (0.92), suggesting slightly lower robustness. Nonetheless, the strong correlation between sobEDAw energies and CBS-extrapolated interaction energies underscores its potential as a reliable energy predictor. The deviation from the ideal slope of 1 in the correlation plots of Figure 6 remains unclear and warrants further investigation, which is beyond the scope of this manuscript.

The percentage contributions of the stabilizing (constructive) components derived from the sobEDAw method are consistent with those obtained from the SAPT method across all studied complexes. For instance, in the case of **Dim-XB**, both the sobEDAw and SAPT approaches show the same order of contributions: *E*_els_(*E*_ele_) > *E*_disp_(*E*_dis_) > *E*_orb_(*E*_ind_) for the absolute values of the stabilizing components. To gain deeper insight into the key contributors responsible for complex stabilization and to better characterize the nature of the NCIs under investigation, the percentage contributions of the constructive components derived from the sobEDAw method (% Δ*E*_els_, % Δ*E*_orb_, and % Δ*E*_disp_) are compared with those from the SAPT approach (% *E*_ele_, % *E*_ind_, and % *E*_dis_), as shown in the last three columns of Table 5 and plotted in Figure 7. It is important to note that since the percentage contributions of the stabilizing components are entirely consistent between the sobEDAw and SAPT approaches (following the same order), to enhance clarity and avoid text congestion, our interpretations will now focus solely on the results from the sobEDAw approach.

**Figure 7:**
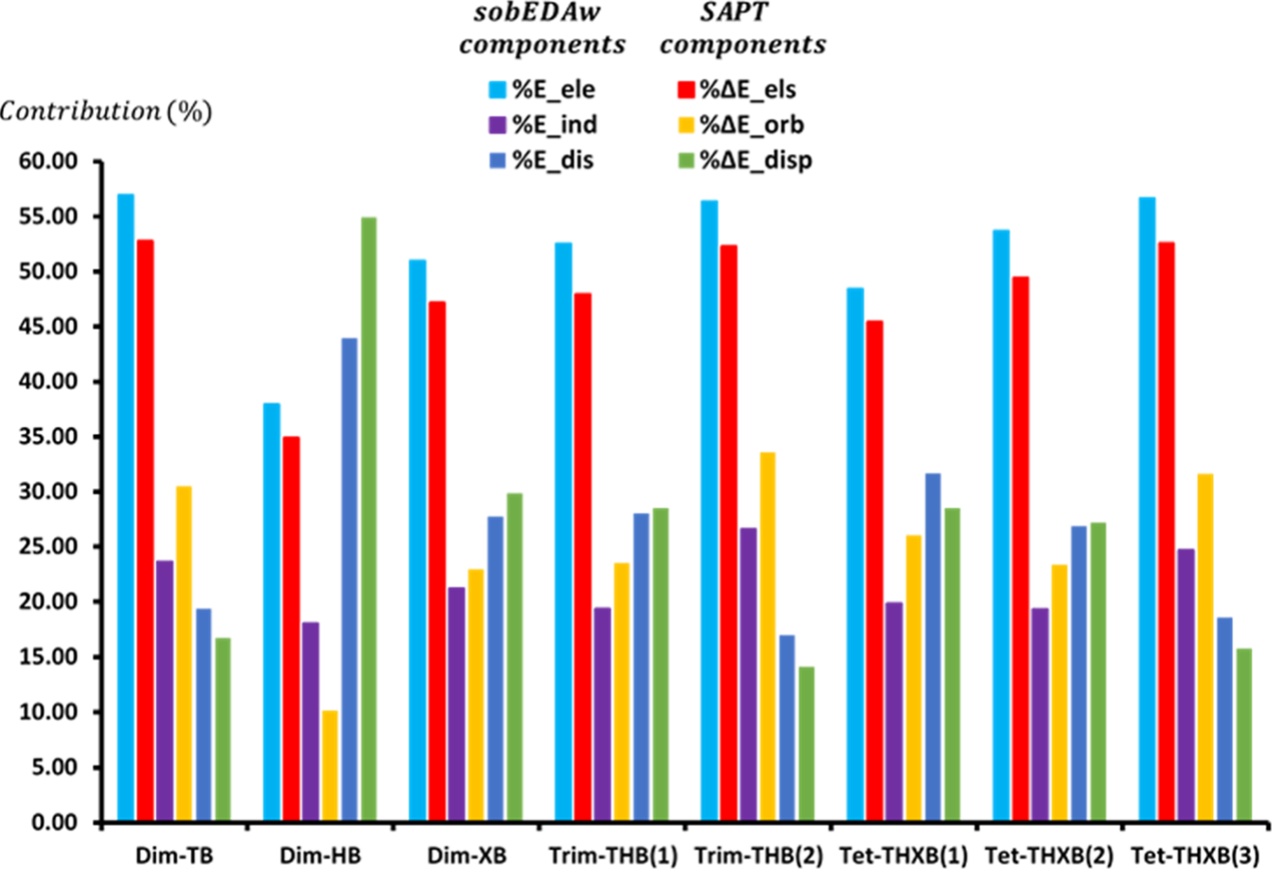
Contribution percentage of the sobEDAw-derived (Δ*E*_els_, Δ*E*_orb_, and Δ*E*_disp_) and gold SAPT-derived (*E*_ele_, *E*_ind_, and *E*_dis_) constructive (stabilizing) components for the NCIs in the complexes considered in Table 5. **Trim-THB­(1)** = **Dim-TB** + **M-2**, **Trim-THB­(2)** = **Dim-HB** + **M-2**, **Tet-THXB­(1)** = **Trim-THB** + **M-2**, **Tet-THXB­(2)** = **Trim-TXB** + **M-2**, **Tet-THXB­(3)** = **Trim-HXB** + **M-2**.

In **Dim-TB**, the primary stabilizing factors are electrostatic interactions (% Δ*E*_els_ = 52.81), alongside significant contributions from orbital interactions (% Δ*E*_orb_ = 30.48) which account for more than half of the electrostatic contribution. As a result, the formation of the Ge···N tetrel bond (TB) in **Dim-TB** from separate **M-1** and **M-2** is primarily driven by electrostatic and orbital interactions, with the role of dispersion interactions being negligible. In **Dim-HB**, dispersion interactions make the largest contribution (% Δ*E*_disp_ = 54.91), but electrostatic interactions, contributing 64% of the dispersion value, also play a key role. This indicates that the O···H hydrogen bond (HB) in **Dim-HB** is dominated by dispersion and electrostatic interactions, while orbital interactions do not play a significant role. This finding aligns with a classification by Emamian et al., who found that very weak hydrogen-bonded dimers with binding energies greater (more positive) than −2.5 kcal·mol^–1^ are primarily stabilized by dispersion and electrostatic interactions.^82^ In **Dim-XB**, the Cl···N halogen bond (XB) is mainly stabilized by electrostatic interactions (% Δ*E*_els_ = 47.21) and dispersion (% Δ*E*_disp_ = 29.85). The bulky nature of the halogen atoms involved in the XB interactions enhances the dispersion contributions in such NCIs.

According to the analyses presented in Scheme 4, both the Ge···N TB and the O···H HB NCIs become stronger in **Trim-THB** compared to their counterparts in **Dim-TB** and **Dim-HB**. The EDA data in the fifth row of Table 5, corresponding to **Trim-THB­(1)** (treated as **Dim-TB** + **M-2**), illustrates the impact of the already-formed TB interaction on the HB interaction. A comparison between the data for **Trim-THB­(1)** and **Dim-HB** (shown in the third row) highlights the stabilizing factors responsible for the stronger HB interaction in **Trim-THB** compared to **Dim-HB**. In **Dim-HB**, dispersion and electrostatic interactions account for 54.91% and 34.94% of the stabilization, respectively, which aligns with expectations for very weak HB NCIs.^82^ In contrast, in **Trim-THB­(1)**, electrostatic interactions remain the largest stabilizing factor (% Δ*E*_els_ = 47.99), while dispersion (% Δ*E*_disp_ = 28.49) and orbital (% Δ*E*_orb_ = 23.52) contributions are nearly equal. These results indicate that, compared to **Dim-HB**, the presence of the Ge···N TB in **Trim-THB­(1)** enhances the O···H HB through increased electrostatic and orbital interactions. The stronger orbital contribution in **Trim-THB­(1)** also suggests that the HB interaction in the trimer exhibits a somewhat more covalent character than in **Dim-HB**.

Similarly, a straightforward comparison between the EDA characteristics of **Dim-TB** (shown in the second row of Table 5) and those of **Trim-THB­(2)** [treated as **Dim-HB** + **M-2**, in the sixth row of Table 5] helps to explain why the Ge···N TB NCI in the trimer is stronger than in **Dim-TB**. In **Dim-TB**, the Ge···N TB is primarily stabilized by electrostatic interactions (% Δ*E*_els_ = 52.81) and orbital interactions (% Δ*E*_orb_ = 30.48). In **Trim-THB­(2)**, the Ge···N TB is similarly supported by electrostatic (% Δ*E*_els_ = 52.32) and orbital (% Δ*E*_orb_ = 33.59) interactions. As shown, while the electrostatic interaction decreases slightly by 0.49% in **Trim-THB­(2)**, the orbital interaction increases by 3.11%. This suggests that the strengthening of the Ge···N TB in **Trim-THB­(2)** is driven by the increase in orbital interactions, which is likely due to the already formed HB NCI in the trimer. Thus, the HB enhances the TB strength through increased orbital contributions. It was also found that while the Ge···N TB and O···H HB interactions in **Tet-THXB** become stronger, the Cl···N XB weakens compared to their respective interactions in **Dim-TB**, **Dim-HB**, and **Dim-XB** (see Scheme 4 and related analyses). As shown, **Tet-THXB** can be treated as **Tet-THXB­(1)** = **Trim-THB** + **M-2**, **Tet-THXB­(2)** = **Trim-TXB** + **M-2**, and **Tet-THXB­(3)** = **Trim-HXB** + **M-2**, with their EDA characteristics summarized in rows seven, eight, and nine of Table 5. Using a similar approach to **Trim-THB**, the mutual impacts of NCIs in **Tet-THXB** can be rationalized. In **Tet-THXB­(1)**, the Cl···N XB is formed via interaction between **Trim-THB**, which already contains TB and HB interactions, and **M-2**. In **Dim-XB**, electrostatic and dispersion interactions account for 47.21% and 29.85%, respectively, of the Cl···N XB stabilization. **In Tet-THXB(1)**, however, these contributions decrease to 45.45% and 28.50%, indicating that the diminutive effect of TB and HB interactions on the XB interaction is mainly due to the reduction in electrostatic and dispersion forces. In **Tet-THXB­(2)**, where the O···H HB is formed through the interaction between **Trim-TXB** (with preexisting TB and XB interactions) and **M-2** (eighth row, Table 5), electrostatic interactions (% Δ*E*_els_ = 49.47%) and dispersion interactions (% Δ*E*_disp_ = 27.18%) are the primary stabilizing factors. In contrast, in **Dim-HB**, the HB interaction is dominated by electrostatics (% Δ*E*_els_ = 34.94%) and dispersion (% Δ*E*_disp_ = 54.91%). This comparison shows that while the TB and XB interactions reduce the dispersion contributions in **Tet-THXB­(2)**, they significantly increase the electrostatic contributions, resulting in a stronger HB interaction in **Tet-THXB­(2)** than in **Dim-HB**, due to the cooperative effects of TB and XB interactions. Finally, the EDA characteristics of **Tet-THXB­(3)**, treated as **Trim-HXB** + **M-2** (last row of Table 5), show that when the TB interaction forms in the presence of preexisting HB and XB interactions, electrostatic (% Δ*E*_els_ = 52.59%) and orbital (% Δ*E*_orb_ = 31.63%) contributions play the primary roles in stabilizing the TB. Comparing these values with those for TB in **Dim-TB** (% Δ*E*_els_ = 52.81% and % Δ*E*_orb_ = 30.48%) reveals that while electrostatic contributions remain largely unchanged from **Dim-TB** to **Tet-THXB­(3)**, the slight increase in orbital contributions accounts for the stronger TB interaction in the tetramer compared to the dimer.

## 4 Conclusion

This comprehensive theoretical study explored the interplay of tetrel (TB), hydrogen (HB), and halogen bonds (XB) in dimers, trimers, and tetramers formed between F_3_GeOCl and HCN. By employing various computational techniques, we demonstrated the cooperative effects between these noncovalent interactions (NCIs). The cooperativity observed in **Trim-THB** and **Tet-THXB** shows that TB and HB interactions mutually reinforce one another, while the presence of HB and TB weakens the XB interaction, resulting in a diminutive effect.

The SAPT and sobEDAw methods provide consistent insights into the stabilizing forces in the complexes studied herein. Electrostatic interactions were found to be the primary stabilizing factor for TB, while dispersion forces significantly contributed to HB and XB stabilization, especially in the presence of the bulky Cl halogen atom. The observed cooperativity enhances the strength of the interactions, particularly the TB and HB, leading to greater stabilization of the complexes.

The correlation between interaction energy and bond critical point (BCP) charge density was nearly perfect, indicating the predictive power of these descriptors in understanding NCIs. These findings offer valuable insights into the fundamental nature of NCIs and their application in molecular design, with potential implications for supramolecular chemistry and crystal engineering.

## Supplementary Material



## Data Availability

All data supporting the findings of this study are included in the Supporting Information. No additional data sets were used for this research.

## Notes

The SI of the present study includes Cartesian coordinates of the fully optimized structure of stationary points located over the PES of complexation processes (PDF)
