# Supplementary material for: Interplay of Tetrel, Hydrogen, and Halogen Bonds in F3GeOCl and HCN Complexes: A Comprehensive Theoretical Study of Dimers, Trimers, and Tetramers
Source: J Phys Chem A. 2025 Jan 28;129(5):1368–85. doi: 10.1021/acs.jpca.4c08102 (PMC12128032; doi:10.1021/acs.jpca.4c08102)
Supplement: Supplementary file 1 [file jp4c08102_si_001.pdf]

Supporting Information (SI)

Interplay of Tetrel, Hydrogen, and Halogen Bonds in F_3GeOCl and HCN Complexes: A Comprehensive Theoretical Study of Dimers, Trimers, and Tetramers

Antonio Frontera[a*] and Saeedreza Emamian[b*]

[a]*Department of Chemistry, Universitat de les Illes Balears, Crta de Valldemossa km 7.5, 07122 Palma de Mallorca (Baleares), SPAIN*

[b]*Department of Chemistry and Biochemistry, Shahrood Branch, Islamic Azad University, Shahrood, Iran.*

Antonio Frontera: toni.frontera@uib.es & ORCID: 0000-0001-7840-2139

Saeedreza Emamian: s_emamian@iau-shahrood.ac.ir; saeedreza_em@yahoo.com & ORCID: 0000-0001-8223-1264

Index

Cartesian coordinates of the MP2/aug-cc-pVDZ fully optimized structure of stationary points located over the PES of complexation processes…………………………………………..S2-S5

Monomer M-1

Ge	-0.55681200	-0.00393100	0.00000000
F	-0.66869300	1.00549800	1.38658400
F	-0.66869200	1.00549900	-1.38658300
F	-1.86057800	-1.12066000	-0.00000100
O	0.91209000	-1.02704800	0.00000000
Cl	2.31193600	0.01936200	0.00000000

Monomer M-2

H	0.00000000	0.00000000	-1.59268800
C	0.00000000	0.00000000	-0.51438300
N	0.00000000	0.00000000	0.66842700

Dimer Dim-TB

Ge	0.11036300	0.20599300	-0.00002400
F	0.24294700	-0.73023600	1.44738000
F	0.24277700	-0.72999100	-1.44758300
F	0.85766200	1.76366400	0.00006500
O	-1.59378700	0.82327700	0.00013700
Cl	-2.70999300	-0.50853600	0.00009800
H	4.62780700	-0.89842600	0.00035400
C	3.58145000	-0.64110700	-0.00002900
N	2.44022700	-0.35982000	-0.00013500

Dimer Dim-HB

Ge	-1.04899300	-0.31501700	0.00234000
F	-1.33720100	-0.18220400	1.68870300
F	-2.28860500	0.53662200	-0.82268300
F	-1.04639300	-1.96398000	-0.46679900
O	0.58963400	0.26215100	-0.45465400
Cl	0.73569000	1.94682400	-0.00801300

H	2.62353700	-0.42512700	-0.18805400
C	3.67192800	-0.65187100	-0.05567500
N	4.81981000	-0.89861000	0.08966900

Dimer Dim-XB

Ge	1.30077000	0.08817300	-0.00012100
F	1.12288200	1.10208500	-1.38001400
F	1.12586000	1.09434900	1.38581200
F	2.88900300	-0.57167400	-0.00345400
O	0.20706500	-1.31762800	-0.00289800
Cl	-1.44734100	-0.71118000	0.00010400
H	-6.13465800	0.90768300	-0.00565200
C	-5.12017800	0.54380500	-0.00168700
N	-4.00862200	0.14517100	0.00285200

Trimer Trim-THB

Ge	-0.61327500	0.04204100	-0.14739100
F	-0.48312500	-0.22370300	1.55659700
F	-1.56057300	1.41330900	-0.60514800
F	-0.60605400	-1.34227300	-1.18385600
O	1.08086700	0.54173100	-0.61064300
Cl	1.55922300	1.96351800	0.26953200
H	-4.76101400	-1.60950900	0.70397500
C	-3.77915000	-1.21453100	0.49892400
N	-2.71031500	-0.78428100	0.27575900
H	2.65857300	-0.72154300	-0.21794000
C	3.50783600	-1.36005000	0.00168300
N	4.43162300	-2.05949400	0.24161500

Trimer Trim-TXB

Ge	0.87233100	-0.32026500	-0.00005400

F	0.72033100	0.62483600	-1.43804200
F	0.72045000	0.62457700	1.43809900
F	2.15920100	-1.47142900	0.00102400
O	-0.49845500	-1.48783200	-0.00084900
Cl	-2.01188300	-0.61004800	-0.00026900
H	4.81794300	2.37571400	0.00019900
C	3.92915100	1.76705300	-0.00000800
N	2.95816600	1.10156600	-0.00031200
H	-6.50785600	1.70538200	0.00029500
C	-5.54609700	1.21972000	0.00035600
N	-4.49147600	0.68677100	0.00042700

Trimer Trim-HXB

Ge	1.49321400	-0.36452300	0.03698300
F	1.34772600	-0.62032000	1.73054500
F	1.65866300	-1.90251400	-0.71104000
F	2.89742700	0.57487400	-0.27309100
O	0.14523200	0.59416000	-0.63920300
Cl	-1.35525200	-0.29503800	-0.35210600
H	-0.64396800	2.52226400	-0.21088900
C	-1.15080300	3.45876200	-0.01917500
N	-1.70523700	4.48266800	0.19045500
H	-5.67462000	-2.46586300	0.41936900
C	-4.71206700	-2.01465900	0.24131000
N	-3.65818600	-1.52013300	0.04625200

Tetramer Tet-THXB

Ge	1.08338600	-0.07945700	-0.20242900
F	0.81273800	-0.06521600	1.50577400
F	1.19457700	-1.65516300	-0.90343200
F	2.11266600	1.15489300	-0.84112800

**O**	**-0.44727200**	**0.60928600**	**-0.89277500**
**Cl**	**-1.81388200**	**-0.38454300**	**-0.43014300**
**H**	**5.20285300**	**-1.42425500**	**1.37324300**
**C**	**4.24031500**	**-1.11404300**	**1.00097600**
**N**	**3.19113700**	**-0.77575900**	**0.59499100**
**H**	**-1.17374200**	**2.37380000**	**-0.16369800**
**C**	**-1.60538200**	**3.29613800**	**0.21333800**
**N**	**-2.07157600**	**4.30333700**	**0.62376600**
**H**	**-6.01672800**	**-2.79143500**	**0.84731600**
**C**	**-5.08666500**	**-2.32557400**	**0.56640600**
**N**	**-4.06755900**	**-1.81361600**	**0.25842300**
